# Mining of Microbial Genomes for the Novel Sources of Nitrilases

**DOI:** 10.1155/2017/7039245

**Published:** 2017-04-12

**Authors:** Nikhil Sharma, Neerja Thakur, Tilak Raj, Savitri, Tek Chand Bhalla

**Affiliations:** ^1^Sub-Distributed Information Centre, Himachal Pradesh University, Summer Hill, Shimla 171005, India; ^2^Department of Biotechnology, Himachal Pradesh University, Summer Hill, Shimla 171005, India

## Abstract

Next-generation DNA sequencing (NGS) has made it feasible to sequence large number of microbial genomes and advancements in computational biology have opened enormous opportunities to mine genome sequence data for novel genes and enzymes or their sources. In the present communication in silico mining of microbial genomes has been carried out to find novel sources of nitrilases. The sequences selected were analyzed for homology and considered for designing motifs. The manually designed motifs based on amino acid sequences of nitrilases were used to screen 2000 microbial genomes (translated to proteomes). This resulted in identification of one hundred thirty-eight putative/hypothetical sequences which could potentially code for nitrilase activity. In vitro validation of nine predicted sources of nitrilases was done for nitrile/cyanide hydrolyzing activity. Out of nine predicted nitrilases,* Gluconacetobacter diazotrophicus*,* Sphingopyxis alaskensis*,* Saccharomonospora viridis*, and* Shimwellia blattae* were specific for aliphatic nitriles, whereas nitrilases from* Geodermatophilus obscurus*,* Nocardiopsis dassonvillei*,* Runella slithyformis*, and* Streptomyces albus* possessed activity for aromatic nitriles.* Flavobacterium indicum* was specific towards potassium cyanide (KCN) which revealed the presence of nitrilase homolog, that is, cyanide dihydratase with no activity for either aliphatic, aromatic, or aryl nitriles. The present study reports the novel sources of nitrilases and cyanide dihydratase which were not reported hitherto by in silico or in vitro studies.

## 1. Introduction

Advancement in the DNA sequencing technologies has led to sequencing of large number of genomes and the enormous sequence data are available in the public domain. The fourth-generation DNA sequencing has made it possible to sequence a bacterial genome within a few hours at a reasonably low cost [1–4]. As of today 5293 prokaryotic and 22 eukaryotic genomes have been completely sequenced and the sequence data are easily accessible in databases such as NCBI, GOLD, and IMG/ER. It is evident from previous studies that not all the gene/protein sequences in the databases are functionally characterized, which make these repositories a rich source for the discovery of novel genes and proteins [5, 6]. Genome mining has emerged as an alternate approach to find novel sources of desired genes/proteins as the conventional screening methods which involve isolation of microbes and their screening for desired products are time consuming, tedious, and cost intensive [7, 8].

Microbial nitrilases are considered to be the most important enzymes in the nitrilase superfamily that find application in the synthesis of fine chemicals, production of some important acids, and drug intermediates and in green chemistry [9–13]. Besides their wide applications nitrilases are prone to certain limitations, for example, their inactivation or inhibition by the acidic product, extremes of pH, temperature, and organic solvent [14, 15]. These limitations are being addressed either by the isolation of microorganisms from the extreme habitat or by enrichment techniques for specific substrate using conventional microbiological procedures [6] prone to limitation as mentioned above. The present communication focuses on in silico screening of publicly available bacterial genomes for nitrilase genes and in vitro validation of the predicted novel sources of nitrilases.

## 2. Material and Methods

### 2.1. Genome Screening Using Homology and Motif Based Approach

Primary screening of microbial genomes (data given as supplementary material in Supplementary Material available online at https://doi.org/10.1155/2017/7039245) was done using homology based approach. Tblastn and blastp were used to screen the sequenced genomes with query sequence to identify the presence and position of similar genes in the genome. Computationally predicted proteins from the bacterial genomes with keyword “nitrilase/cyanide dihydratase” were also downloaded using advanced search options in the IMG/ER database. Sequences with low (30%) and high similarity (80%) were discarded. Nitrilase gene in contigs showing the presence of nitrilase homologs was downloaded from IMG/ER. GenMark S tool was used to predict the ORFs in each contig, and the output was downloaded selecting protein sequence as output option. Amino acid sequences less than 100 amino acids were considered to be as false positive (FP) and were discarded. Small amino acid sequence database was created which was further subjected to local blast, to confirm the presence of nitrilase homolog in the contigs of the individual genome.

On the other hand, protein based manually designed motifs (MDMs) were used to screen the bacterial genome to search for the presence of conserved motifs using MAST (Motif Alignment and Search Tool) at MEME (Multiple Em for Motif Elicitation) suite. The motifs used are already described in our previous communication [12]. Motifs identified in sequences less than hundred amino acids were rejected, considered to be false positive (FP). Sequences above 100 amino acids were taken to be as true positive (TP).

### 2.2. Study of Physiochemical Properties and Phylogenetic Analysis of Predicted Nitrilases

Physiochemical data of the in silico predicted nitrilases were generated from the ProtParam software using ExPASy server and compared to the values deduced from the previous nitrilase study [16]. Some important physiochemical properties such as number of amino acids, molecular weight (kda), isoelectric point (pI), computing pI/Mw and the atomic compositions, values of instability index, aliphatic index, and grand average of hydropathicity (GRAVY) were calculated. A comparative chart was drawn between previously characterized and predicted nitrilases.

An output file of multiple aligned sequences using Clustal W for both previously characterized and predicted nitrilases was used to generate the Neighbor Joining (NJ) tree using MEGA 6 version. Phylogenetic tree was generated in order to predict the sequences as aliphatic or aromatic with previously characterized nitrilases.

### 2.3. Nitrilase Activity Assay

Culture of some of the bacteria predicted to have nitrilase gene* (Shimwellia blattae*,* Runella slithyformis*,* Geodermatophilus obscurus*,* Nocardiopsis dassonvillei*,* Streptomyces albus*,* Flavobacterium indicum*,* Saccharomonospora viridis*,* Sphingopyxis alaskensis*, and* Gluconacetobacter diazotrophicus)* was procured from Microbial Type Culture Collection (MTCC); Chandigarh* Escherichia coli* BL21 (DE3) from Invitrogen was used as negative control as this organism does not have nitrilase gene. These cultures were grown in the laboratory using different media (Table 1) for the production of nitrilase activity following the procedures described earlier [17–19]. Nitrilase activity was assayed in 1.0 mL reaction mixture containing nitrile as substrate (1–10 mM) and 0.1 mL resting cells. After 30 min of incubation at 30°C the reaction was quenched with 0.1 M HCl and the amount of ammonia released was estimated using nitrilase assay, that is, modified phenate-hypochlorite method described by Dennett and Blamey [20]. One unit of nitrilase activity was defined as the amount of enzyme required to release 1 *μ*mole of ammonia per min under the assay conditions.

## 3. Results

### 3.1. Genome Screening Using Conserved Motifs and Homology Search

As many as 138 candidate sequences were identified using tblastn and blastp at IMG/ER on both gene and protein level. Identification of potentially coding nitrilase genes was done using homology based approach (blastp and tblastn) allowing the identification of nitrilase sequences. To identify newer sources of nitrilases, candidate sequences bearing unassigned functions (hypothetical, uncharacterized, or putative) were selected from the translated genomes (Table 2). The identified sequences shared 30–50% sequence identity to biochemically characterized* Rhodococcus rhodochrous* J1 nitrilase which was taken as query sequence. Catalytic residues were found to be conserved in all the predicted proteins. Nine predicted and translated sequences were further chosen for their in silico and in vitro validation based on the manually designed motifs (MDMs) (Tables 3 and 4) identified from previous study [12].

### 3.2. Physiochemical Parameters and Phylogenetic Analysis

In silico identified nitrilases were analyzed for their physiochemical properties using ProtParam, an online tool at the ExPASy proteomic server. The selected candidates values for various properties were found to be very much similar to those with earlier published data by Sharma and Bhalla [16] as mentioned in Table 5. Average values deduced for aliphatic and aromatic nitrilases from earlier characterized proteins were taken as standard for the comparison of a predicted set of nitrilase. The values of the same were found to be very much similar to those with earlier published data by Sharma and Bhalla [16] as mentioned in Table 5. The total number of amino acids ranged from 260 amino acids* (Nocardiopsis dassonvillei)* to 342 amino acids* (Shimwellia blattae)* with different molecular weight. Isoelectric point ranged between 4.8 and 5.8 which is found to be closer to the consensus value, that is, the average data value from previously characterized aliphatic or aromatic nitrilases.

**Figure 1:**
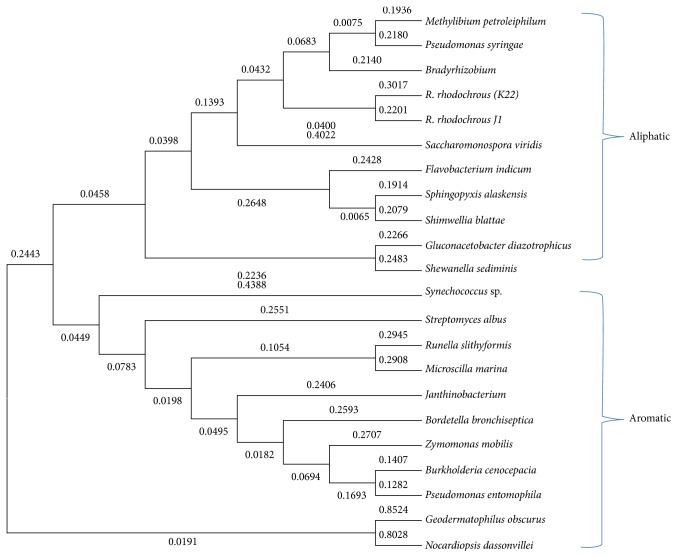
Neighbor Joining (NJ) method differentiating characterized and in silico predicted as aliphatic and aromatic nitrilases.

Neighbor Joining (NJ) tree using MEGA 6 shows the phylogenetic analysis with in silico predicted sequences from completely sequenced microbial genomes with that of previously characterized nitrilase sequences. They were distinguished either as aliphatic or aromatic according to their position in the phylogenetic tree (Figure 1).

### 3.3. In Vitro Validation of Some In Silico Predicted Nitrilases

To validate for nitrile transforming activity of nine predicted novel sources of nitrilases, these were tested against common aliphatic, aromatic, and aryl nitriles and potassium cyanide (KCN).* Gluconacetobacter diazotrophicus*,* Sphingopyxis alaskensis*,* Saccharomonospora viridis*, and* Shimwellia blattae* were found to be more specific for aliphatic nitriles. On the other hand,* Geodermatophilus obscurus*,* Nocardiopsis dassonvillei*,* Runella slithyformis*, and* Streptomyces albus* exhibited nitrilase activity for aromatic nitriles.* Flavobacterium indicum* was the only organism which showed no activity for either aliphatic, aromatic, or aryl nitriles but was specific towards the degradation of the potassium cyanide (KCN) (Table 6). On the other hand, negative control, that is,* Escherichia coli* BL21 (DE3), showed no activity for any of the nitriles/substrates tested.

## 4. Discussion

Annotation of sequenced genomes to identify new genes has become integral part of the research in bioinformatics [21–24]. The present investigation has revealed some novel sources of nitrilases. Homology and conserved motif approach screened microbial genomes and proteins predicted as nitrilase or cyanide dihydratase or carbon-nitrogen hydrolase in 138 prokaryotic bacterial genomes. Manually designed motifs (MDMs) also differentiated the in silico predicted nitrilases as aliphatic or aromatic [12] as the designed motifs are class specific. All the four motifs identified were uniformly conserved throughout the two sets of aliphatic and aromatic nitrilases as mentioned in Table 4.

The sequences belonged to the nitrilase superfamily, showing the presence of the catalytic triad Glu (E), Lys (K), and Cys (C) to be conserved throughout. Phylogenetic analysis using the MEGA 6.0 version for the aliphatic and aromatic set of protein sequences revealed two major clusters. Neighbor Joining (NJ) tree used for phylogenetic analysis revealed that in silico predicted proteins (this study) and previously identified nitrilases as aliphatic and aromatic [16] were found to be grouped in their respective clusters (Figure 1).

Aliphaticity and aromaticity of in silico predicted and characterized nitrilases were differentiated based on their physiochemical properties. The physicochemical properties of the predicted set of nitrilase were deduced using the ProtParam subroutine of Expert Protein Analysis System (ExPASy) from the proteomic server of the Swiss Institute of Bioinformatics (SIB), in order to predict aromaticity or aliphaticity. Several of the parameters (number of amino acids, molecular weight, number of negatively charged residues, extinction coefficients, and grand average of hydropathicity) listed in Table 5 are closer to the consensus values reported for aromatic and aliphatic nitrilases, supporting that the predicted set of nitrilase has aromatic or aliphatic substrate specificity (Table 5).

In silico predictions were verified by in vitro validation of the predicted proteins. Common nitriles (aliphatic, aromatic, and aryl nitriles) and potassium cyanide (KCN) were tested to check for the nitrile/cyanide transforming ability of the predicted proteins. Out of nine predicted proteins eight were found active for different nitriles, whereas* Flavobacterium indicum* was found to hydrolyze toxic cyanide (KCN) into nontoxic form (Table 6). The present approach contributed to finding novel sources of desired nitrilase from microbial genome database.

## 5. Conclusion

Genome mining for novel sources of nitrilases has predicted 138 sources for nitrilases. In vitro validation of the selected nine predicted sources of nitrilases for nitrile/cyanide hydrolyzing activity has furthered the scope of genome mining approaches for the discovery of novel sources of enzymes.

## Supplementary Material

List of organisms with completely sequenced genomes avaliable at NCBI and IMG/ER.

## Figures and Tables

**Table 1:** Composition of various media used to cultivate procured strains for nitrilase production.

Name of the organism	MTCC number	Composition (gL^−1^)	pH	Growth temperature
*Shimwellia blattae* ATCC 29907	4155	Beef extract: 1.0 g Yeast extract: 2.0 g Peptone: 5.0 g NaCl: 5.0 g Agar: 15.0 g	7.0–7.5	37°C

*Runella slithyformis* ATCC 29530	9504	Glucose: 1.0 g Peptone: 1.0 g Yeast extract: 1.0 g Agar: 15.0 g Glucose: 4.0 g	7.0–7.5	26°C

*Geodermatophilus obscurus* DSM 43160	4040	Yeast extract: 4.0 g Malt extract: 10.0 g CaCO_3_: 2.0 g Agar: 12.0 g	7.2–7.5	28°C

*Nocardiopsis dassonvillei* DSM 43111	1411	Yeast extract: 4.0 g Malt extract: 1.0 Glucose: 4.0 g Agar: 20.0 g	7.2–7.4	28°C

*Streptomyces albus* J1074	1138	Yeast extract: 4.0 g Malt extract: 1.0 g Glucose: 4.0 g Agar: 20.0 g	7.2–7.4	25°C

*Flavobacterium indicum* DSM 17447	6936	Tryptic soy broth with agar (TSBA-100)	7.3–7.5	30°C

*Saccharomonospora viridis* ATCC 15386	320	Yeast extract: 4.0 g Malt extract: 1.0 g Glucose: 4.0 g Agar: 20.0 g	7.2–7.4	45°C

*Sphingopyxis alaskensis* DSM 13593	7504	Beef extract: 1.0 g Yeast extract: 2.0 g Peptone: 5.0 g NaCl: 5.0 g Agar: 15.0 g	7.0–7.5	30°C

*Gluconacetobacter diazotrophicus* ATCC 49037	1224	Yeast extract: 5.0 g Peptone: 3.0 g Mannitol: 25.0 g Agar: 15.0 g	7.0–7.3	28°C

*Escherichia coli* BL21 (DE3)^**∗**^	—	Yeast extract: 5.0 g NaCl: 10.0 g Casein enzymatic hydrolysate: 10.0 g	7.0–7.5	37°C

^**∗**^Negative control.

**Table 2:** Prediction of ORFs length in the individual scaffold for prediction of coding sequence for nitrilase using IMG/ER.

Name of organism	Scaffold or genome length (bp) with accession number	Total number of ORF's predicted in scaffold of complete genome	Predicted coding region for nitrilase	Number of base-pairs
*Acaryochloris marina* MBIC11017	NC_009925 (6503724 bp)	152	200001–200999	999

*Acetobacter pasteurianus* IFO 3283-32	AP011157 (191443 bp)	120	174107–173133	974

*Achromobacter xylosoxidans* A8	NC_014640 (7013095 bp)	406	200001–200960	960

*Acidovorax avenae avenae* ATCC 19860	NC_015138 (5482170 bp)	188	201035–200001	1035

*Acidothermus cellulolyticus* 11B	NC_008578 (2443540 bp)	403	200001–201131	1131

*Acidaminococcus fermentans* VR4	NC_013740 (2329769 bp)	293	200924–200001	924

*Alcanivorax dieselolei* B5	CP003466 (4928223 bp)	343	200001–200981	981

*Arthrobacter aurescens* TC1	NC_008711 (4597686 bp)	385	200001–200930	930

*Azorhizobium caulinodans* ORS 571	NC_009937 (5369772 bp)	262	89665–88580	1083

*Azospirillum *sp. B510	NC_013854 (3311395 bp)	402	200001–200921	921

*Bacillus pumilus* SAFR-032	NC_009848 (3704465 bp)	73	201026–200001	1026

*Bradyrhizobium japonicum* USDA 110	NC_004463 (9105828 bp)	387	200001–200966	966

*Bradyrhizobium *sp. BTAi1	NC_009485 (8264687 bp)	392	201146–200001	1146

*Bradyrhizobium *sp. ORS278	NC_009445 (7456587 bp)	395	201041–200001	1041

*Brevibacillus brevis* NBRC 100599	NC_012491 (6296436 bp)	182	200001–200960	960

*Flavobacterium indicum* GPTSA100-9	HE774682 (2993089 bp)	317	200001–200981	981

*Saccharomonospora viridis* P101	NC_013159 (4308349 bp)	315	200001–200996	996

*Sphingopyxis alaskensis* DSM13593	NC_008048 (3345170 bp)	387	200001–201017	1017

*Burkholderia cenocepacia* J2315	NC_011000 (3870082 bp)	393	199944–201050	1050

*Burkholderia glumae* BGR1	NC_012720 (141067 bp)	154	47491–48477	1017

*Burkholderia gladioli* BSR3	NC_015376 (3700833 bp)	338	200001–201014	1014

*Burkholderia phymatum*	NC_010623 (2697374 bp)	375	199971–201023	1023

*Burkholderia phytofirmans*	NC_010681 (4467537 bp)	357	200001–201035	1035

*Burkholderia *sp. CCGE1002	NC_014119 (1282816 bp)	280	72013–73041	1020

*Burkholderia* sp. CCGE1003	NC_014540 (2966498 bp)	344	200019–201041	1022

*Burkholderia vietnamiensis *G4	NC_009254 (1241007 bp)	436	199986–201023	1037

*Burkholderia xenovorans* LB400	NC_007951 (4895836 bp)	396	200001–200996	996

*Caulobacter* sp. K31	NC_010335 (233649 bp)	219	180936–181871	935

*Chlorobium phaeobacteroides* BS1	NC_010831 (2736403 bp)	382	200001–200936	936

*Clostridium difficile* 630	NC_009089 (4290252 bp)	364	200001–200927	927

*Clostridium difficile* CD196	NC_013315 (4110554 bp)	308	200001–200927	927

*Clostridium difficile* R20291	NC_013316 (4191339 bp)	329	200001–200927	927

*Clostridium kluyveri* NBRC 12016	NC_011837 (3896121 bp)	442	200001–200930	957

*Clostridium kluyveri* ATCC 8527	NC_009706 (3964618 bp)	491	200001–200930	930

*Conexibacter woesei* DSM 14684	NC_013739 (6359369 bp)	388	200001–200942	942

*Cupriavidus necator* ATCC 17699	NC_008313 (4052032 bp)	318	200001–201017	1017

*Cupriavidus necator* ATCC 43291	NC_015726 (3872936 bp)	318	200001–201017	1017

*Cyanobium gracile* ATCC 27147	Cyagr_Contig81 (3342364 bp)	405	200001–200999	999

*Deinococcus deserti* (strain VCD115)	NC_012529 (314317 bp)	269	200001–200951	951

*Deinococcus peraridilitoris* DSM 19664	Deipe_Contig72.1 (3881839 bp)	412	200001–200951	951

*Desulfomonile tiedjei* ATCC 49306	Desti_Contig107.1 (6500104 bp)	379	200001–201029	1029

*Dickeya zeae* Ech1591	NC_012912 (4813854 bp)	194	200001–200927	927

*Erwinia billingiae* Eb661	NC_014305 (169778 bp)	194	87964–88965	1001

*Erythrobacter litoralis* HTCC2594	NC_007722 (3052398 bp)	411	200001–200969	969

*Flavobacterium indicum* DSM 17447	HE774682 (2993089 bp)	317	200001–200981	981

*Frateuria aurantia* ATCC 33424	Fraau_Contig24.1 (3603458 bp)	366	200001–200924	924

*Geobacillus* sp. Y4.1MC1	NC_014650 (3840330 bp)	434	200001–200966	966

*Geobacillus thermoglucosidasius* C56-YS93	NC_015660 (3893306 bp)	446	200001–200966	966

*Geodermatophilus obscurus* DSM 43160	NC_013757 (5322497 bp)	244	54102–54884	783

*Gluconacetobacter diazotrophicus* ATCC 49037	NC_010125 (3944163 bp)	333	200001–200960	960

*Haliangium ochraceum* DSM 14365	CP002175 (2309262 bp)	377	200001–200957	957

*Halanaerobium praevalens* ATCC 33744	NC_013440 (9446314 bp)	262	200001–200999	999

*Hyphomicrobium* sp. MC1	NC_015717 (4757528 bp)	392	200001–200984	984

*Janthinobacterium* sp. Marseille	NC_009659 (4110251 bp)	398	200001–201068	1068

*Jannaschia* sp. CCS1	NC_007802 (4317977 bp)	382	200001–201026	1026

*Maricaulis maris* MCS10	NC_008347 (3368780 bp)	392	200001–200933	933

*Methylobacterium extorquens* CM4	NC_011758 (380207 bp)	211	7191–8267	1077

*Methylobacterium extorquens* ATCC 14718	NC_012811 (1261460 bp)	436	200001–201077	1077

*Methylobacterium extorquens* DM4	NC_012988 (5943768 bp)	378	200001–200918	918

*Methylobacterium extorquens *PA1	NC_010172 (5471154 bp)	354	200001–201110	1110

*Methylomonas methanica* MC09	Contig38 (5051681 bp)	402	200001–200996	996

*Methylobacterium nodulans* ORS2060	NC_011892 (487734 bp)	425	200001–201116	1116

*Methylobacterium populi* ATCC BAA-705	NC_010725 (5800441 bp)	193	61617–62693	1077

*Methylibium petroleiphilum* PM1	NC_008825 (4044195 bp)	364	200001–201074	1074

*Methylobacterium radiotolerans* ATCC 27329	NC_010505 (6077833 bp)	377	200001–201077	1077

*Methylocella silvestris* BL2	NC_011666 (4305430 bp)	439	199971–201029	1029

*Mycobacterium intracellulare* ATCC 13950	CP003322 (5402402 bp)	383	199938–200897	897

*Mycobacterium liflandii* 128FXT	CP003899 (6208955 bp)	405	200001–201059	1059

*Mycobacterium rhodesiae* NBB3	MycrhN_Contig54.1 (6415739 bp)	267	200001–200957	957

*Mycobacterium smegmatis* ATCC 700084	CP001663 (6988208 bp)	377	200001–200978	978

*Natranaerobius thermophilus* ATCC BAA-1301	NC_010718 (3165557 bp)	387	200001–200930	930

*Nocardia farcinica* IFM 10152	NC_006361 (6021225 bp)	390	198993–199811	818

*Nocardiopsis dassonvillei* DSM 43111	NC_014211 (775354 bp)	353	201134–200001	843

*Oligotropha carboxidovorans* ATCC 49405	CP002826 (3595748 bp)	372	200001–201065	1065

*Pantoea* sp. At-9b	NC_014839 (394054 bp)	349	114577–115581	1005

*Peptoniphilus duerdenii* ATCC BAA-1640	NZ_AEEH01000050 (96694 bp)	80	52942–53863	921

*Photorhabdus asymbiotica* ATCC 43949	NC_012962 (5064808 bp)	338	200001–201050	1050

*Pirellula staleyi* ATCC 27377	NC_013720 (6196199 bp)	338	200001–200909	909

*Polaromonas naphthalenivorans* CJ2	NC_008781 (4410291 bp)	389	200001–201041	1062

*Polaromonas *sp. JS666	NC_007948 (5200264 bp)	398	200001–200942	942

*Pseudomonas syringae* pv. *lachrymans* M302278PT	Lac106_115287.20 (115287 bp)	107	47704–48747	1043

*Pseudoalteromonas atlantica* ATCC BAA-1087	NC_008228 (5187005 bp)	397	200001–200921	921

*Pseudomonas aeruginosa* P7-L633/96	Ga0060317_132 (369634 bp)	270	91986–92801	816

*Pseudomonas brassicacearum* NFM421	NC_015379 (6843248 bp)	377	200001–201026	1026

*Pseudomonas* sp. TJI-51	AEWE01000051 (6502 bp)	05	1482–2498	1017

*Pseudomonas fluorescens* Pf-5	NC_007492 (6438405 bp)	349	200001–200924	924

*Pseudomonas fluorescens* SBW25	NC_012660 (6722539 bp)	376	200043–200930	888

*Pseudomonas mendocina* NK-01	NC_015410 (5434353 bp)	376	200001–200883	883

*Pseudomonas syringae* pv. tomato DC 3000	PSPTOimg_DC3000 (6397126 bp)	377	200001–201011	1011

*Pseudomonas syringae* pv.* syringae* B728a	NC_007005 (6093698 bp)	196	8233–9231	999

*Pseudoxanthomonas suwonensis*11-1	NC_014924 (3419049 bp)	362	200001–200885	885

*Pseudonocardia dioxanivorans* ATCC 55486	CP002593 (7096571 bp)	386	200001–201008	1008

*Ralstonia solanacearum* GMI1000	NC_003295 (3716413 bp)	343	200001–201032	1032

*Rhizobium hainanense* CCBAU 57015	Ga0061100_113 (148344 bp)	146	61240–62280	1040

*Rhizobium leguminosarum *bv. *Viciae *3841	NC_008380 (5057142 bp)	397	200001–201047	1047

*Rhizobium leguminosarum* bv. *trifolii* WSM1325	NC_012850 (4767043 bp)	210	18450–19442	993

*Rhodopseudomonaspalustris* TIE-1	NC_011004 (5744041 bp)	387	199980–201050	1070

*Rhodopseudomonas palustris* DX-1	NC_014834 (5404117 bp)	390	200001–200954	954

*Rubrobacter xylanophilus* DSM 9941	NC_008148 (3225748 bp)	385	200001–201080	1080

*Ruegeria pomeroyi* ATCC 700808	NC_006569 (491611 bp)	308	118859–119893	1035

*Runella slithyformis* ATCC 29530	Unknown (6568739 bp)	362	200001–200933	933

*Saccharothrix espanaensis* ATCC 51144	HE804045 (9360653 bp)	347	200001–201020	1020

*Saccharomonospora viridis* ATCC 15386	NC_013159 (4308349 bp)	315	200001–200996	996

*Shewanella halifaxensis* HAW-EB4	NC_010334 (5226917 bp)	337	200001–200945	945

*Shewanella pealeana* ATCC 700345	NC_009901 (5174581 bp)	333	200001–200945	945

Shewanella sediminis HAW-EB3	NC_009831 (5517674 bp)	337	200001–200954	954

*Shewanella violacea* JCM 1017	NC_014012 (4962103 bp)	307	200001–200936	936

*Shewanella woodyi* ATCC 51908	NC_010506 (5935403 bp)	327	200001–201005	1005

*Shimwellia blattae* ATCC 29907	EBLc (4158725 bp)	376	200001–201029	1029

*Singulisphaera acidiphila* ATCC 1392	Sinac_Contig49.1 (9629675 bp)	337	200001–201014	1014

*Sorangium cellulosum *Soce56	NC_010162 (13033779 bp)	329	200001–201029	1029

*Sphingopyxis alaskensis* DSM 13593	NC_008048 (3345170 bp)	387	200001–201017	1016

*Sphaerobacter thermophilus* DSM 20745	NC_013524 (1252731 bp)	335	200097–201092	995

*Sphingomonas wittichii* RW1	NC_009511 (5382261 bp)	354	200001–201026	1026

*Spirosoma linguale* ATCC 33905	NC_013730 (8078757 bp)	339	200001–200906	906

*Starkeya novella* ATCC 8093	NC_007604 (2695903 bp)	402	200001–201005	1005

*Streptomyces albus *J1074	CP004370 (6841649 bp)	252	1635309–1636256	948

*Synechococcus elongatus* PCC 7942	NC_007604 (2695903 bp)	402	200001–201005	1005

*Syntrophobacter fumaroxidans* DSM 10017	NC_008554 (4990251 bp)	337	200001–200987	987

*Synechococcus *sp. ATCC 27264	NC_010475 (3008047 bp)	431	200001–201008	1008

*Synechococcus elongatus* *PCC 6301*	NC_006576 (2696255 bp)	402	200001–201005	1005

*Synechococcus *sp. PCC 7002	NC_010475 (3008047 bp)	431	200001–201008	1008

*Synechococcus *sp. WH8102	NC_005070 (2434428 bp)	537	200001–201017	1017

*Synechocystis* sp.	CP003265 (3569561 bp)	371	200001–201026	1026

*Synechocystis *sp. PCC 6803	NC_017052 (3570103 bp)	374	200001–201026	1026

*Terriglobus roseus* KBS 63	Terro_Contig51.1 (5227858 bp)	354	200001–200873	873

*Tistrella mobilis* KA081020-065	CP003239 (1126962 bp)	379	200001–201077	1077

*Variovorax paradoxus* (strain EPS)	NC_014931 (6550056 bp)	360	200001–201035	1035

*Variovorax paradoxus* S110	NC_012791 (5626353 bp)	420	200001–201053	1053

*Verminephrobacter eiseniae* EF01-2	NC_008786 (5566749 bp)	337	200001–200987	1020

*Zobellia galactanivorans* DSM 12802	FG20DRAFT (5340688 bp)	331	200001–200951	951

*Zymomonas mobilis* subsp.* Mobilis* ATCC 10988	NZ_ACQU01000006 (113352 bp)	113	82520–83509	990

**Table 3:** Manually designed motifs (MDMs) for aliphatic and aromatic nitrilases showing the presence of essential catalytic triad (**E**, **K,** and **C**).

Nitrilases	Manually designed motif
Aliphatic	[FL]-[ILV]-[AV]-F-P-**E**-[VT]-[FW]-[IL]-P-[GY]-Y-P-[WY]
	R-R-**K**-[LI]-[KRI]-[PA]-T-[HY]-[VAH]-E-R
	**C**-W-E-H-[FLX]-[NQ]-[PT]-L
	[VA]-A-X-[AV]-Q-[AI]-X-P-[VA]-X-[LF]-[SD]

Aromatic	[ALV]-[LV]-[FLM]-P-**E**-[AS]-[FLV]-[LV]-[AGP]-[AG]-Y-P
	[AGN]-[KR]-H-R-**K**-L-[MK]-P-T-[AGN]-X-E-R
	**C**-W-E-N-[HY]-M-P-[LM]-[AL]-R-X-X-[ML]-Y
	A-X-E-G-R-C-[FW]-V-[LIV]

**Table 4:** Aliphatic and aromatic nitrilase motif patterns with bold letter depicting catalytic center (**E**, **K**, and **C**) in predicted nitrilases.

Nitrilases	Manually Designed motif	1	2	3	4	5	6	7	8	9
Aliphatic	**[FL]-[ILV]-[AV]-F-P-E-[VT]-[FW]-[IL]-P-[GY]-Y-P-[WY]**	A-F-P**-E-**V-F-V-P-A-Y-P-Y	F-P-**E**-L-W-L-P-G-Y-P-I-F	F-P-**E-**V-F-I-S-G-Y-P-Y-W-N-W				F-P-**E**-V-F-I-A-G-Y	F-P-**E**-T-F-V-P-Y-Y-P-Y	
	**R-R-K-[LI]-[KRI]-[PA]-T-[HY]-[VAH]-E-R**	L-R-R-**K-L**-V-P-T-W	R-R-**K**-L-K-P-T-H-V-E-R	R**-K**-L-V-P-T-W-A-E-K-L-T				R-H-R-**K**-L-V-P-T-W-A-E-R	R-R-**K**-I-T-P-T-Y-H-E-R	
	**C-W-E-H-[FLX]-[NQ]-[PT]-L**	**C-**G-E-N-T-N-T-L-A	**C**-A-E-N-M-Q-P-L	**C**-G-E-N-T-N-T-L-A				**C**-G-E-N-T-N-T-L-A-R-F-S	**C**-W-E-H-Y-N-P-L-	
	**[VA]-A-X-[AV]-Q-[AI]- X- P-[VA]-X-[LF]-[SD]**	V-A-A-V-Q-A-A-P-V-F-L-D-P	V-A-S-V-Q-A-E	V-Q-T-A-P-V-F-L-N-V-E				A-A-V-Q-A-A-P-V-F-L	A-A-V-Q-I-S-P-V-L-	

Aromatic	**[ALV]-[LV]-[FLM]-P-E-[AS]-[FLV]-[LV]-[AGP]-[AG]-Y-P**				F-Q-**E**-V-F-N-A	P-**E**-S-F-I-P-C-Y-P-R-G	F-P-**E**-A-F-L-G-T-Y-P			S-**E**-T-F-S-T-G
	**[AGN]-[KR]-H-R-K-L-[MK]-P-T-[AGN]-X-E-R**				R-**K**-H-H-I-P-Q-V	H-R-**K**-L-K-P-T-G-L-E-R	H-R-**K-**V-M-P-T-G-A-E-R			R-**K**-L-H-P-F-T
	**C-W-E-N-[HY]-M-P-[LM]-[AL]-R-X-X-[ML]-Y**				**C**-Y-D-R-H	**C**-W-E-N-Y-M-P-L-A-R-M	**C**-W-E-N-Y-M-P-L-L-R-A			**C**-Y-D-L-R-F-A
	**A-X-E-G-R-C-[FW]-V-[LIV]**				A-H-L-W-K-L-E	A-L-E-G-R-C-F-V-L-A	A-L-E-G-R-C-W-V			A-I-E-N-Q-A-Y-V

**Table 5:** Comparison of physiochemical properties of aliphatic, aromatic, and predicted nitrilase from the average consensus values reported by Sharma and Bhalla [16].

Parameters	Average value for aliphatic	Average value for aromatic	1	2	3	4	5	6	7	8	9
Number of amino acids	352.2	309.8	338.0	331.0	326.0	260.0	280.0	310.0	315.0	342.0	319.0

Molecular weight (Da)	38274.0	33693.5	36154.9	36491.2	36364.7	27903.3	31464.1	34938.1	33821.5	37472.7	34678.7

Theoretical pI	5.5	5.5	5.0	4.9	6.2	5.2	5.6	5.4	4.8	5.4	5.8

NCR^*∗*^	41.7	35.8	41.0	44.0	40.0	32.0	36.0	43.0	43.0	41.0	39.0

PCR^*∗*^	30.3	29.2	26.0	25.0	37.0	21.0	27.0	34.0	29.0	30.0	32.0

Extinction coefficients (M^−1^ cm^−1^) at 280 nm	50213.3	43975.0	45295.0	33015.0	43890.0	35200.0	62465.0	53400.0	47900.0	38305.0	31775.0

Instability index	41.2	38.5	30.1	52.5	27.0	27.7	28.6	39.6	46.6	36.6	38.5

Aliphatic index	89.40	89.90	94.1	87.9	93.6	81.1	76.0	90.9	86.2	92.8	89.3

Grand average of hydropathicity (GRAVY)	00.10	00.01	0.027	−0.17	−0.14	−0.051	−0.283	−0.109	0.045	−0.052	−0.002

NCR^*∗*^: negatively charged residues; PCR^*∗*^: positively charged residues.

**Table 6:** Nitrilase activity^*∗*^ of in silico predicted microbial sources of nitrilases assayed using common aliphatic, aromatic, aryl aliphatic, and KCN as substrate.

Organisms	Substrates								
	Valeronitrile	Benzonitrile	Mandelonitrile	Isobutyronitrile	Adiponitrile	2-Cyanopyridine	Propionitrile	Acrylonitrile	KCN
*Streptomyces albus* J1074	0.0015	0.0027	ND	0.0014	ND	ND	ND	ND	ND

*Nocardiopsis dassonvillei* DSM 43111	ND	0.0040	0.0024	ND	ND	ND	ND	ND	ND

*Geodermatophilus obscurus* DSM 43160	ND	0.0043	0.0021	ND	ND	ND	ND	ND	ND

*Shimwellia blattae* ATCC 29907	0.0028	ND	0.0016	0.0019	ND	ND	ND	ND	ND

*Runella slithyformis* ATCC 29530	ND	0.0297	0.0152	0.0095	ND	ND	0.0169	ND	ND

*Gluconacetobacter diazotrophicus* ATCC 49037	ND	0.0016	0.0020	0.0051	0.0048	ND	ND	ND	ND

*Sphingopyxis alaskensis* DSM 13593	ND	0.00073	ND	0.0024	0.00075	ND	ND	ND	ND

*Saccharomonospora viridis* ATCC 15386	ND	ND	ND	0.0030	ND	ND	ND	ND	ND

*Flavobacterium indicum* DSM 17447	ND	ND	ND	ND	ND	ND	ND	ND	0.25

*Escherichia coli* BL21 (DE3)^*∗∗*^	ND	ND	ND	ND	ND	ND	ND	ND	ND

^**∗**^Expressed as *µ*mole of ammonia released/min/mg dcw under the assay conditions; ND = not detected; ^*∗∗*^negative control.

## References

[1] Seffernick J. L., Samanta S. K., Louie T. M., Wackett L. P., Subramanian M. (2009). Investigative mining of sequence data for novel enzymes: a case study with nitrilases. *Journal of Biotechnology*.

[2] Land M., Hauser L., Jun S. (2015). Insights from 20 years of bacterial genome sequencing. *Functional & Integrative Genomics*.

[3] Yadav D. (2015). Relevance of bioinformatics in the era of omics driven research. *Journal of Next Generation Sequencing & Applications*.

[4] Feng Y., Zhang Y., Ying C., Wang D., Du C. (2015). Nanopore-based fourth-generation DNA sequencing technology. *Genomics, Proteomics & Bioinformatics*.

[5] Van Lanen S. G., Shen B. (2006). Microbial genomics for the improvement of natural product discovery. *Current Opinion in Microbiology*.

[6] Luo X. J., Yu H. L., Xu J. H. (2012). Genomic data mining: an efficient way to find new and better enzymes. *Enzyme Engineering*.

[7] De Sousa-Pereira P., Amado F., Abrantes J., Ferreira R., Esteves P. J., Vitorino R. (2013). An evolutionary perspective of mammal salivary peptide families: cystatins, histatins, statherin and PRPs. *Archives of Oral Biology*.

[8] Adrio J. L., Demain A. L. (2014). Microbial enzymes: tools for biotechnological processes. *Biomolecules*.

[9] Raj J., Singh N., Prasad S., Seth A., Bhalla T. C. (2007). Bioconversion of benzonitrile to benzoic acid using free and agar entrapped cells of *Nocardia globerula* NHB-2. *Acta Microbiologica et Immunologica Hungarica*.

[10] Rao M. A., Scelza R., Scotti R., Gianfreda L. (2010). Role of enzymes in the remediation of polluted environments. *Journal of Soil Science and Plant Nutrition*.

[11] Kaul P., Asano Y. (2012). Strategies for discovery and improvement of enzyme function: state of the art and opportunities. *Microbial Biotechnology*.

[12] Sharma N. N., Sharma M., Bhalla T. C. (2012). *Nocardia globerula* NHB-2 nitrilase catalysed biotransformation of 4-cyanopyridine to isonicotinic acid. *AMB Express*.

[13] Bhatia S. K., Mehta P. K., Bhatia R. K., Bhalla T. C. (2014). Optimization of arylacetonitrilase production from *Alcaligenes* sp. MTCC 10675 and its application in mandelic acid synthesis. *Applied Microbiology and Biotechnology*.

[14] Gong J.-S., Lu Z.-M., Li H., Shi J.-S., Zhou Z.-M., Xu Z.-H. (2012). Nitrilases in nitrile biocatalysis: recent progress and forthcoming research. *Microbial Cell Factories*.

[15] Ramteke P. W., Maurice N. G., Joseph B., Wadher B. J. (2013). Nitrile-converting enzymes: an eco-friendly tool for industrial biocatalysis. *Biotechnology and Applied Biochemistry*.

[16] Sharma N., Bhalla T. C. (2012). Motif design for nitrilases. *Journal of Data Mining in Genomics & Proteomics*.

[17] Sharma N., Kushwaha R., Sodhi J. S., Bhalla T. C. (2009). In silico analysis of amino acid sequences in relation to specificity and physiochemical properties of some microbial nitrilases. *Journal of Proteomics and Bioinformatics*.

[18] Bhalla T. C., Miura A., Wakamoto A., Ohba Y., Furuhashi K. (1992). Asymmetric hydrolysis of *α*-aminonitriles to optically active amino acids by a nitrilase of *Rhodococcus rhodochrous* PA-34. *Applied Microbiology and Biotechnology*.

[19] Vejvoda V., Kubáč D., Davidová A. (2010). Purification and characterization of nitrilase from *Fusarium solani* IMI196840. *Process Biochemistry*.

[20] Dennett G. V., Blamey J. M. (2016). A new thermophilic nitrilase from an antarctic hyperthermophilic microorganism. *Frontiers in Bioengineering and Biotechnology*.

[21] Fawcett J. K., Scott J. E. (1960). A rapid and precise method for the determination of urea. *Journal of Clinical Pathology*.

[22] Friedberg I. (2006). Automated protein function prediction—the genomic challenge. *Briefings in Bioinformatics*.

[23] Armengaud J. (2009). A perfect genome annotation is within reach with the proteomics and genomics alliance. *Current Opinion in Microbiology*.

[24] Poptsova M. S., Gogarten J. P. (2010). Using comparative genome analysis to identify problems in annotated microbial genomes. *Microbiology*.

