# Supplementary material for: Mining of Microbial Genomes for the Novel Sources of Nitrilases
Source: Biomed Res Int. 2017 Apr 12;2017:7039245. doi: 10.1155/2017/7039245 (PMC5405348; doi:10.1155/2017/7039245)
Supplement: Supplementary file 1 — List of organisms with completely sequenced genomes avaliable at NCBI and IMG/ER. [file 7039245.f1.pdf]

S.No.	Name of the organism
1.	Acaryochloris marina
2.	Accumulibacter phosphatis (strain UW-1)
3.	Acetohalobium arabaticum (strain ATCC 49924 / DSM 5501 / Z-7288)
4.	Acetobacterium woodii (strain ATCC 29683 / DSM 1030 / JCM 2381 / KCTC 1655)
5.	Acholeplasma laidlawii (strain PG-8A)
6.	Acidovorax citrulli (strain AAC00-1)
7.	Acinetobacter sp. (strain ADP1)
8.	Acidovorax avenae (strain ATCC 19860 / DSM 7227 / JCM 20985 / NCPPB 1011)
9.	Acinetobacter baumannii (strain 1656-2)
10.	Acinetobacter baumannii (strain AB307-0294)
11.	Acinetobacter baumannii (strain AB0057)
12.	Acinetobacter baumannii (strain ACICU)
13.	Acinetobacter baumannii (strain TCDC-AB0715)
14.	Acinetobacter baumannii (strain SDF)
15.	Acinetobacter baumannii (strain ATCC 17978 / NCDC KC 755)
16.	Acinetobacter baumannii (strain AYE)
17.	Acidothermus cellulolyticus (strain ATCC 43068 / 11B)
18.	Acidobacterium capsulatum (strain ATCC 51196 / DSM 11244 / JCM 7670)
19.	Acidiphilium cryptum (strain JF-5)
20.	Acinetobacter calcoaceticus (strain PHEA-2)
21.	Acidithiobacillus caldus (strain SM-1)
22.	Acidovorax ebreus (strain TPSY)
23.	Acidithiobacillus ferrooxidans (strain ATCC 23270 / DSM 14882 / NCIB 8455)
24.	Acidithiobacillus ferrooxidans ATCC 53993
25.	Acidimicrobium ferrooxidans (strain DSM 10331 / JCM 15462 / NBRC 103882 / ICP)
26.	Acidaminococcus fermentans
27.	Acidaminococcus intestini (strain RyC-MR95)
28.	Acidiphilium multivorum (strain DSM 11245 / JCM 8867 / AIU301)
29.	Acinetobacter sp. (strain JCM 1667 / KCTC 23045 / DR1)
30.	Acidovorax sp. (strain JS42)
31.	Acidobacterium sp

32.	Actinoplanes missouriensis (strain ATCC 14538)
33.	Actinosynnema mirum (strain ATCC 29888 / DSM 43827 / NBRC 14064 / IMRU 3971)
34.	Actinobacillus pleuropneumoniae serotype 5b (strain L20)
35.	Actinobacillus pleuropneumoniae serotype 7 (strain AP76)
36.	Actinobacillus pleuropneumoniae serotype 3 (strain JL03)
37.	Actinoplanes sp. (strain ATCC 31044 / CBS 674.73 / SE50/110)
38.	Actinobacillus succinogenes (strain ATCC 55618 / 130Z)
39.	Advenella kashmirensis WT001
40.	Aequorivita sublithincola (strain DSM 14238 / LMG 21431 / ACAM 643 / 9-3)
41.	Aeromonas hydrophila subsp. hydrophila (strain ATCC 7966 / NCIB 9240)
42.	AERS4 Aeromonas salmonicida (strain A449)
43.	Aerococcus urinae (strain ACS-120-V-Col10a)
44.	Aeromonas veronii (strain B565)
45.	Aggregatibacter aphrophilus (strain NJ8700)
46.	Agrobacterium radiobacter (strain K84 / ATCC BAA-868)
47.	Akkermansia muciniphila (strain ATCC BAA-835)
48.	Alcanivorax borkumensis (strain SK2 / ATCC 700651 / DSM 11573)
49.	Alcanivorax dieselolei (strain DSM 16502 / CGMCC 1.3690 / B-5)
50.	Alkalilimnicola ehrlichei (strain MLHE-1)
51.	Alicyclobacillus acidocaldarius subsp. acidocaldarius (strain ATCC 27009 / DSM 446 / 104-1A)
52.	Alicyclobacillus acidocaldarius (strain Tc-4-1)
53.	Alicycliphilus denitrificans (strain JCM 14587 / BC)
54.	Alicycliphilus denitrificans (strain DSM 14773 / CIP107495 / K601)
55.	Alistipes finegoldii (strain DSM 17242 / JCM 16770 /AHN 2437 / CCUG 46020 / CIP 107999)
56.	Aliivibrio salmonicida (strain LFI1238)
57.	Alkaliphilus metalliredigens (strain QYMF)
58.	Alkaliphilus oremlandii (strain OhILAs)
59.	Allochromatium vinosum (strain ATCC 17899 / DSM 180 /NBRC 103801 / D)
60.	Alteromonas macleodii (strain Balearic Sea AD45)

61.	Alteromonas macleodii (strain DSM 17117 / Deep ecotype)
62.	Alteromonas macleodii (strain English Channel 673)
63.	Alteromonas macleodii (strain Black Sea 11)
64.	Alteromonas sp. (strain SN2)
65.	Aminobacterium colombiense (strain DSM 12261 / ALA-1)
66.	Ammonifex degensii (strain DSM 10501 / KC4)
67.	Amoebophilus asiaticus (strain 5a2)
68.	Amphibacillus xylanus (strain ATCC 51415 / DSM 6626 /JCM 7361 / LMG 17667 / NBRC 15112 / Ep01
69.	Amycolatopsis mediterranei (strain U-32)
70.	Amycolicicoccus subflavus (strain DSM 45089 /DQS3-9A1)
71.	Anabaena cylindrica (strain ATCC 27899 / PCC 7122)
72.	Anaplasma centrale (strain Israel)
73.	Anaeromyxobacter dehalogenans (strain 2CP-1 / ATCC BAA-258)
74.	Anaeromyxobacter dehalogenans (strain 2CP-C)
75.	ANADF Anaeromyxobacter sp. (strain Fw109-5)
76.	Anaerobaculum mobile (strain ATCC BAA-54 / DSM 13181 /NGA)
77.	Anaplasma marginale (strain Florida)
78.	Anaplasma marginale (strain St. Maries)
79.	Anaerococcus prevotii (strain ATCC 9321 / DSM 20548 / JCM 6508 / PC1)
80.	Anaplasma phagocytophilum (strain HZ)
81.	Anaeromyxobacter sp. (strain K)
82.	naerolinea thermophila (strain DSM 14523 / JCM 11388 / NBRC 100420 / UNI-1)
83.	Anabaena variabilis (strain ATCC 29413 / PCC 7937)
84.	Anoxybacillus flavithermus (strain DSM 21510 / WK1)
85.	Aquifex aeolicus (strain VF5)
86.	Arcobacter butzleri (strain RM4018)
87.	Arcanobacterium haemolyticum (strain ATCC 9345 / DSM 20595 / NBRC 15585 / NCTC 8452 / 11018)
88.	Arcobacter nitrofigilis (strain ATCC 33309 / DSM 7299

	/ LMG 7604 / NCTC 12251 / CI)
89.	Aromatoleum aromaticum (strain EbN1)
90.	Arthrobacter arilaitensis (strain DSM 16368 / CIP 108037 / JCM 13566 / Re117)
91.	Arthrobacter aurescens (strain TC1)
92.	Arthrobacter chlorophenolicus (strain A6 / ATCC 700700 / DSM 12829 / JCM 12360)
93.	Arthrobacter phenanthrenivorans (strain DSM 18606 /JCM 16027 / LMG 23796 / Sphe3)
94.	Arthrobacter sp. (strain FB24)
95.	Arthromitus sp. (strain SFB-mouse-Japan)
96.	Asticcacaulis excentricus (strain ATCC 15261 / DSM4724 / VKM B-1370 / CB 48)
97.	Atopobium parvulum (strain ATCC 33793 / DSM 20469 /JCM 10300 / VPI 0546)
98.	Aster yellows witches'-broom phytoplasma (strain AYWB)
99.	Azorhizobium caulinodans (strain ATCC 43989 / DSM 5975/ ORS 571)
100.	Azospirillum lipoferum (strain 4B)
101.	Azobacteroides pseudotrichonymphae genomovar. CFP2
102.	Azospirillum sp. (strain B510)
103.	Azoarcus sp. (strain BH72)
104.	Azospira oryzae (strain ATCC BAA-33 / DSM 13638 / PS)
105.	Azotobacter vinelandii (strain DJ / ATCC BAA-1303)
106.	Bacillus atrophaeus (strain 1942)
107.	Bacillus amyloliquefaciens (strain FZB42)
108.	Bacillus anthracis (strain A0248)
109.	Bacillus anthracis (strain CDC 684 / NRRL 3495)
110.	Bacillus thuringiensis (strain Al Hakam)
111.	Bacillus cereus var. anthracis (strain CI)
112.	Bacillus anthracis
113.	Bacillus amyloliquefaciens (strain ATCC 23350 / DSM 7/ BCRC 11601 / NBRC 15535 / NRRL B-14393)
114.	Bacillus cereus (strain AH820)
115.	Bacillus cereus (strain ATCC 10987)
116.	Bacillus cereus (strain G9842)
117.	Bacillus cereus (strain 03BB102)

118.	Bacillus cereus (strain B4264)
119.	Bacillus coagulans (strain 2-6)
120.	Bacillus cereus (strain AH187)
121.	Bacillus cellulosilyticus (strain ATCC 21833 / DSM2522 / FERM P-1141 / JCM 9156 / N-4)
122.	Bacillus cereus subsp. cytotoxis (strain NVH 391-98)
123.	Bacillus cereus (strain Q1)
124.	Bacillus cereus (strain ATCC 14579 / DSM 31)
125.	Bacillus cereus (strain ZK / E33L)
126.	Bacteroides fragilis (strain 638R)
127.	Bacteroides fragilis ATCC 25285
128.	Bacteroides fragilis (strain YCH46)
129.	Bacillus halodurans (strain ATCC BAA-125 / DSM 18197 /FERM 7344 / JCM 9153 / C-125)
130.	Bacillus thuringiensis subsp. konkukian (strain 97-27)
131.	Bacillus selenitireducens (strain ATCC 700615 / DSM15326 / MLS10)
132.	Bacillus licheniformis (strain DSM 13 / ATCC 14580)
133.	Bacillus megaterium (strain DSM 319)
134.	Bacillus megaterium (strain ATCC 12872 / QMB1551)
135.	Bacillus megaterium (strain ATCC 14581 / DSM 32 / JCM 2506 / NBRC 15308 / NCIMB 9376 / NCTC 10342 / VKM B-512)
136.	Bacteriovorax marinus (strain ATCC BAA-682 / DSM 15412/ SJ)
137.	Bacillus pumilus (strain SAFR-032)
138.	Bacillus pseudofirmus (strain OF4)
139.	Bacillus subtilis subsp. spizizenii (strain ATCC 23059/ NRRL B-14472 / W23)
140.	Bacteroides salanitronis (strain DSM 18170 / JCM 13567/ BL78)
141.	Bacillus clausii (strain KSM-K16)
142.	Bacillus subtilis (strain BSn5)
143.	Bacillus subtilis (strain 168)
144.	Bacillus thuringiensis subsp. finitimus (strainYBT-020)
145.	Bacillus thuringiensis (strain BMB171)
146.	Bacillus tusciae (strain DSM 2912 / NBRC 15312 / T2)
147.	Bacteroides helcogenes (strain ATCC 35417 / DSM 20613/ JCM 6297 / P 36-108)
148.	Bacteroides thetaiotaomicron (strain ATCC 29148 / DSM2079 / NCTC 10582 / E50 / VPI-

	5482)
149.	Bacteroides vulgatus (strain ATCC 8482 / DSM 1447 /NCTC 11154)
150.	Bacillus weihenstephanensis (strain KBAB4)
151.	Bartonella australis (strain Aust/NH1)
152.	Bartonella bacilliformis (strain ATCC 35685 / KC583)
153.	Bartonella clarridgeiae (strain CIP 104772 / 73)
154.	Bartonella grahamii (strain as4aup)
155.	Bartonella henselae (strain ATCC 49882 / Houston 1)
156.	Bartonella quintana (strain Toulouse)
157.	Bartonella tribocorum (strain CIP 105476 / IBS 506)
158.	Bartonella vinsonii subsp. berkhoffii (strain Winnie)
159.	Baumannia cicadellinicola subsp. Homalodisca coagulata
160.	Bdellovibrio bacteriovorus (strain ATCC 15356 / DSM50701 / NCIB 9529 / HD100)
161.	Beijerinckia indica subsp. indica (strain ATCC 9039 /DSM 1715 / NCIB 8712)
162.	Belliella baltica (strain DSM 15883 / CIP 108006 / LMG21964 / BA134)
163.	Beutenbergia cavernae (strain ATCC BAA-8 / DSM 12333 /NBRC 16432)
164.	Bifidobacterium animalis subsp. lactis (strain AD011)
165.	Bifidobacterium adolescentis (strain ATCC 15703 / DSM 20083 / NCTC 11814 / E194a)
166.	Bifidobacterium animalis subsp. lactis (strain V9)
167.	Bifidobacterium breve (strain ACS-071-V-Sch8b)
168.	Bifidobacterium bifidum (strain PRL2010)
169.	Bifidobacterium bifidum (strain S17)
170.	Bifidobacterium breve (strain NCIMB 8807 / UCC2003)
171.	Bifidobacterium dentium (strain ATCC 27534 / DSM 20436/ JCM 1195 / Bd1)
172.	Bifidobacterium longum subsp. infantis (strain 157F)
173.	Bifidobacterium longum subsp. longum (strain ATCC 15707 / DSM 20219 / JCM 1217 / NCTC 11818 / E194b)
174.	Bifidobacterium animalis subsp. lactis (strain Bl-04 /DGCC2908 / RB 4825 / SD5219)
175.	Bifidobacterium longum (strain DJO10A)
176.	Bifidobacterium longum subsp. longum (strain JDM301)
177.	Bifidobacterium longum subsp. longum (strain BBMN68)
178.	Bifidobacterium longum (strain NCC 2705)

179.	Bifidobacterium longum subsp. infantis (strain ATCC 15697 / DSM 20088 / JCM 1222 / NCTC 11817 / S12)
180.	Blattabacterium sp. subsp. Blattella germanica (strain Bge)
181.	Blastococcus saxobsidens (strain DD2)
182.	Blattabacterium sp. subsp. Periplaneta Americana (strain Blochmannia floridanus BPLAN)
183.	Blochmannia pennsylvanicus (strain BPEN)
184.	Blochmannia vafer (strain BVAF)
185.	Bordetella avium (strain 197N)
186.	BORAP Borrelia afzelii (strain PKo)
187.	Borrelia bissettii (strain DN127)
188.	Borrelia burgdorferi (strain JD1)
189.	Bordetella bronchiseptica (strain MO149)
190.	Borrelia burgdorferi (strain N40)
191.	Bordetella bronchiseptica (strain ATCC BAA-588 / NCTC 13252 / RB50)
192.	Borrelia burgdorferi (strain ATCC 35210 / B31 / CIP 102532 / DSM 4680)
193.	Borrelia burgdorferi (strain ZS7)
194.	Borrelia crocidurae (strain Achema)
195.	Borrelia duttonii (strain Ly)
196.	Borrelia garinii (strain PBi)
197.	Borrelia hermsii (strain DAH)
198.	Bordetella pertussis (strain ATCC 9797 / DSM 5571 /NCTC 10739 / 18323)
199.	Bordetella parapertussis (strain 12822 / ATCC BAA-587/ NCTC 13253)
200.	Bordetella parapertussis (strain Bpp5)
201.	Bordetella pertussis (strain CS)
202.	Bordetella petrii (strain ATCC BAA-461 / DSM 12804 /CCUG 43448)
203.	Bordetella pertussis (strain Tohama I / ATCC BAA-589 /NCTC 13251)
204.	Borrelia recurrentis (strain A1)
205.	Candidatus Chloracidobacterium thermophilum B
206.	Candidatus Chloracidobacterium thermophilum B
207.	Candidatus Cloacamonas acidaminovorans.
208.	Candidatus Desulfococcus oleovorans Hxd3

209.	Candidatus Desulforudis audaxviator MP104C
210.	Candidatus Hamiltonella defensa 5AT Acyrthosiphon pisum
211.	Candidatus Hodgkinia cicadicola Dsem
212.	Candidatus Kinetoplastibacterium blastocrithidii ex Strigomonas
213.	Candidatus Kinetoplastibacterium blastocrithidii TCC012E
214.	Candidatus Kinetoplastibacterium crithidii ex Angomonas deanei ATCC 30255
215.	Candidatus Kinetoplastibacterium crithidii TCC036E
216.	Candidatus Kinetoplastibacterium desouzaii TCC079E
217.	Candidatus Kinetoplastibacterium galatii TCC219
218.	Candidatus Kinetoplastibacterium oncopeltii TCC290E
219.	Candidatus Korarchaeum cryptofilum OPF8
220.	Candidatus Liberibacter asiaticus str. gxpsy
221.	Candidatus Liberibacter asiaticus str. psy62
222.	Candidatus Liberibacter solanacearum CLso-ZC1
223.	Candidatus Methanoregula boonei 6A8
224.	Candidatus Methanosphaerula palustris E1-9c
225.	Candidatus Midichloria mitochondrii IricVA
226.	Candidatus Moranella endobia PCIT
227.	Candidatus Mycoplasma haemolamae str. Purdue
228.	Candidatus Nitrosopumilus koreensis AR1
229.	Candidatus Nitrosopumilus sp. AR2
230.	Candidatus Nitrososphaera gargensis Ga9.2
231.	Candidatus Nitrospira defluvii
232.	Candidatus Pelagibacter sp. IMCC9063
233.	Candidatus Pelagibacter ubique HTCC1062
234.	Candidatus Phytoplasma australiense
235.	Candidatus Phytoplasma mali
236.	Candidatus Portiera aleyrodidarum BT-B
237.	Candidatus Portiera aleyrodidarum BT-B-HRs
238.	Candidatus Portiera aleyrodidarum BT-Q-AWR
239.	Candidatus Portiera aleyrodidarum BT-QVLC
240.	Candidatus Rickettsia amblyommii str. GAT-30V

241.	Candidatus Riesia pediculicola USDA
242.	Candidatus Ruthia magnifica str. Cm Calyptogena magnifica
243.	Candidatus Sulcia muelleri CARI
244.	Candidatus Sulcia muelleri DMIN
245.	Candidatus Sulcia muelleri GWSS
246.	Candidatus Sulcia muelleri SMDSEM
247.	Candidatus Tremblaya princeps PCIT
248.	Candidatus Tremblaya princeps PCVAL
249.	Candidatus Uzinura diaspidicola str. ASNER
250.	Candidatus Vesicomyosocius okutanii HA
251.	Candidatus Zinderia insecticola CARI
252.	Capnocytophaga canimorsus Cc5
253.	Capnocytophaga ochracea DSM 7271
254.	Carboxydothermus hydrogenoformans Z-2901
255.	Cardinium endosymbiont cEper1 of Encarsia pergandiella
256.	Carnobacterium maltaromaticum LMA28
257.	Carnobacterium sp. 17-4
258.	Catenulispora acidiphila DSM 44928
259.	Caulobacter crescentus
260.	Caulobacter crescentus NA1000
261.	Caulobacter segnis ATCC 21756
262.	Caulobacter sp. K31
263.	Cellulomonas fimi ATCC 484
264.	Cellulomonas flavigena DSM 20109
265.	Cellulophaga algicola DSM 14237
266.	Cellulophaga lytica DSM 7489
267.	Cellvibrio gilvus ATCC 13127
268.	Cellvibrio japonicus Ueda107
269.	Cenarchaeum symbiosum A
270.	Chamaesiphon minutus PCC 6605
271.	Chitinophaga pinensis DSM 2588
272.	Chlamydia muridarum

273.	Chlamydia psittaci 84/55
274.	Chlamydia psittaci CP3
275.	Chlamydia psittaci GR9
276.	Chlamydia psittaci M56
277.	Chlamydia psittaci MN
278.	Chlamydia psittaci NJ1
279.	Chlamydia psittaci VS225
280.	Chlamydia psittaci WC
281.	Chlamydia psittaci WS/RT/E30
282.	Chlamydia trachomatis Jali20
283.	Chlamydia trachomatis 434/Bu
284.	Chlamydia trachomatis A/HAR-13
285.	Chlamydia trachomatis A2497
286.	Chlamydia trachomatis A2497
287.	Chlamydia trachomatis A2497 sero A
288.	Chlamydia trachomatis B/TZ1A828/OT
289.	Chlamydia trachomatis D-EC
290.	Chlamydia trachomatis D-LC
291.	Chlamydia trachomatis D/UW-3/CX
292.	Chlamydia trachomatis E/11023
293.	Chlamydia trachomatis E/150
294.	Chlamydia trachomatis E/SW3
295.	Chlamydia trachomatis F/SW4
296.	Chlamydia trachomatis F/SW5
297.	Chlamydia trachomatis G/11074
298.	Chlamydia trachomatis G/11222
299.	Chlamydia trachomatis G/9301
300.	Chlamydia trachomatis G/9768
301.	Chlamydia trachomatis IU824
302.	Chlamydia trachomatis L2b/UCH-1/proctitis
303.	Chlamydia trachomatis L2c
304.	Chlamydia trachomatis Sweden2

305.	Chlamydophila abortus S26/3
306.	Chlamydophila caviae GPIC
307.	Chlamydophila felis Fe/C-56
308.	Chlamydophila pecorum E58
309.	Chlamydophila pneumoniae AR39
310.	Chlamydophila pneumoniae CWL029
311.	Chlamydophila pneumoniae J138
312.	Chlamydophila pneumoniae LPCoLN
313.	Chlamydophila pneumoniae TW-183
314.	Chlamydophila psittaci 01DC11
315.	Chlamydophila psittaci 02DC15
316.	Chlamydophila psittaci 08DC60
317.	Chlamydophila psittaci 6BC
318.	Chlamydophila psittaci 6BC VR-125
319.	Chlamydophila psittaci C19/98
320.	Chlamydophila psittaci Mat116
321.	Chlamydophila psittaci RD1
322.	Chlorobaculum parvum NCIB 8327
323.	Chlorobium chlorochromatii CaD3
324.	Chlorobium limicola DSM 245
325.	Chlorobium phaeobacteroides BS1
326.	Chlorobium phaeobacteroides DSM 266
327.	Chlorobium tepidum TLS
328.	Chloroflexus aggregans DSM 9485
329.	Chloroflexus aurantiacus J-10-fl
330.	Chloroflexus sp. Y-400-fl
331.	Chloroherpeton thalassium ATCC 35110
332.	Chromobacterium violaceum ATCC 12472
333.	Chromohalobacter salexigens DSM 3043
334.	Chroococcidiopsis thermalis PCC 7203
335.	Citrobacter koseri ATCC BAA-895
336.	Citrobacter rodentium ICC168

337.	Clavibacter michiganensis ssp. michiganensis NCPPB 382
338.	Clavibacter michiganensis ssp. sepedonicus
339.	Clostridiales genomosp. BVAB3 str. UPII9-5
340.	Clostridium acetobutylicum ATCC824
341.	Clostridium acetobutylicum DSM 1731
342.	Clostridium acetobutylicum EA 2018
343.	Clostridium acidurici 9a
344.	Clostridium beijerinckii NCIMB 8052
345.	Clostridium botulinum A str. ATCC 19397
346.	Clostridium botulinum A str. ATCC 3502
347.	Clostridium botulinum A str. Hall
348.	Clostridium botulinum A2 str. Kyoto
349.	Clostridium botulinum A3 str. Loch Maree
350.	Clostridium botulinum B str. Eklund 17B
351.	Clostridium botulinum B1 str. Okra
352.	Clostridium botulinum BKT015925
353.	Clostridium botulinum Ba4 str. 657
354.	Clostridium botulinum E3 str. Alaska E43
355.	Clostridium botulinum F str. 230613
356.	Clostridium botulinum F str. Langeland
357.	Clostridium botulinum H04402 065
358.	Clostridium cellulolyticum H10
359.	Clostridium cellulovorans 743B
360.	Clostridium clariflavum DSM 19732
361.	Clostridium difficile 630
362.	Clostridium difficile CD196
363.	Clostridium difficile R20291
364.	Clostridium kluyveri DSM 555
365.	Clostridium kluyveri NBRC 12016
366.	Clostridium lentocellum DSM 5427
367.	Clostridium ljungdahlii ATCC 49587
368.	Clostridium novyi NT

369.	Clostridium perfringens
370.	Clostridium perfringens ATCC 13124
371.	Clostridium perfringens SM101
372.	Clostridium phytofermentans ISDg
373.	Clostridium saccharolyticum WM1
374.	Clostridium saccharoperbutylacetonicum N1-4HMT
375.	Clostridium sp. BNL1100
376.	Clostridium sp. SY8519
377.	Clostridium stercorarium ssp. stercorarium DSM 8532
378.	Clostridium tetani E88
379.	Clostridium thermocellum ATCC 27405
380.	Clostridium thermocellum DSM 1313
381.	Collimonas fungivorans Ter331
382.	Colwellia psychrerythraea 34H
383.	Comamonas testosteroni CNB-2
384.	Conexibacter woesei DSM 14684
385.	Coprothermobacter proteolyticus DSM 5265
386.	Coraliomargarita akajimensis DSM 45221
387.	Corallococcus coralloides DSM 2259
388.	Coriobacterium glomerans PW2
389.	Corynebacterium aurimucosum ATCC 700975
390.	Corynebacterium callunae DSM 20147
391.	Corynebacterium diphtheriae
392.	Corynebacterium diphtheriae 241
393.	Corynebacterium diphtheriae 31A
394.	Corynebacterium diphtheriae BH8
395.	Corynebacterium diphtheriae C7 beta
396.	Corynebacterium diphtheriae CDCE 8392
397.	Corynebacterium diphtheriae HC01
398.	Corynebacterium diphtheriae HC02
399.	Corynebacterium diphtheriae HC03
400.	Corynebacterium diphtheriae HC04

401.	Corynebacterium diphtheriae INCA 402
402.	Corynebacterium diphtheriae PW8
403.	Corynebacterium diphtheriae VA01
404.	Corynebacterium efficiens YS-314
405.	Corynebacterium glutamicum ATCC 13032 Bielefeld
406.	Corynebacterium glutamicum ATCC 13032 Kitasato
407.	Corynebacterium glutamicum K051 strain ATCC 13032
408.	Corynebacterium glutamicum R
409.	Corynebacterium halotolerans YIM 70093 = DSM 44683
410.	Corynebacterium jeikeium K411
411.	Corynebacterium kroppenstedtii DSM 44385
412.	Corynebacterium pseudotuberculosis 1/06-A
413.	Corynebacterium pseudotuberculosis 1002
414.	Corynebacterium pseudotuberculosis 258
415.	Corynebacterium pseudotuberculosis 267
416.	Corynebacterium pseudotuberculosis 42/02-A
417.	Corynebacterium pseudotuberculosis C231
418.	Corynebacterium pseudotuberculosis CIP 52.97
419.	Corynebacterium pseudotuberculosis Cp162
420.	Corynebacterium pseudotuberculosis FRC41
421.	Corynebacterium pseudotuberculosis I19
422.	Corynebacterium pseudotuberculosis P54B96
423.	Corynebacterium pseudotuberculosis PAT10
424.	Corynebacterium resistens DSM 45100
425.	Corynebacterium ulcerans 0102
426.	Corynebacterium ulcerans 809
427.	Corynebacterium ulcerans BR-AD22
428.	Corynebacterium urealyticum DSM 7109
429.	Corynebacterium urealyticum DSM 7111
430.	Corynebacterium variabile DSM 44702
431.	Coxiella burnetii CbuG_Q212
432.	Coxiella burnetii CbuK_Q154

433.	Coxiella burnetii Dugway 7E9-12
434.	Coxiella burnetii RSA 331
435.	Coxiella burnetii st. RSA 493
436.	Crinalium epipsammum PCC 9333
437.	Croceibacter atlanticus HTCC2559
438.	Cronobacter sakazakii ES15
439.	Cronobacter sakazakii Sp291
440.	Cronobacter turicensis
441.	Cryptobacterium curtum DSM 15641
442.	Cupriavidus taiwanensis 1
443.	Cupriavidus taiwanensis 2
444.	Cyanobacteria bacterium Yellowstone A-Prime
445.	Cyanobacteria bacterium Yellowstone B-Prime
446.	Cyanobacterium UCYN-A
447.	Cyanobacterium aponinum PCC 10605
448.	Cyanobacterium stanieri PCC 7202
449.	Cyanobium gracile PCC 6307
450.	Cyanothece sp. ATCC 51142
451.	Cyanothece sp. PCC 7424
452.	Cyanothece sp. PCC 7425
453.	Cyanothece sp. PCC 7822
454.	Cyanothece sp. PCC 8801
455.	Cyanothece sp. PCC 8802
456.	Cyclobacterium marinum DSM 745
457.	Cycloclasticus sp. P1
458.	Cylindrospermum stagnale PCC 7417
459.	Cytophaga hutchinsonii ATCC 33406
460.	Dactylococcopsis salina PCC 8305
461.	Dechloromonas aromatica RCB
462.	Dechlorosoma suillum PS
463.	Deferribacter desulfuricans SSM1
464.	Dehalobacter sp. CF

465.	Dehalobacter sp. DCA
466.	Dehalococcoides ethenogenes 195
467.	Dehalococcoides mccartyi BTF08
468.	Dehalococcoides mccartyi DCMB5
469.	Dehalococcoides sp. BAV1
470.	Dehalococcoides sp. CBDB1
471.	Dehalococcoides sp. GT
472.	Dehalococcoides sp. VS
473.	Dehalogenimonas lykanthroporepellens BL-DC-9
474.	Deinococcus deserti VCD115
475.	Deinococcus geothermalis DSM 11300.
476.	Deinococcus gobiensis I-0
477.	Deinococcus maricopensis DSM 21211
478.	Deinococcus peraridilitoris DSM 19664
479.	Deinococcus proteolyticus MRP
480.	Deinococcus radiodurans R1
481.	Delftia acidovorans SPH-1
482.	Delftia sp. Cs1-4
483.	Denitrovibrio acetiphilus DSM 12809
484.	Desulfarculus baarsii DSM 2075
485.	Desulfatibacillum alkenivorans AK-01
486.	Desulfitobacterium dehalogenans ATCC 51507
487.	Desulfitobacterium dichloroeliminans LMG P-21439
488.	Desulfitobacterium hafniense DCB-2
489.	Desulfitobacterium hafniense Y51
490.	Desulfobacca acetoxidans DSM 11109
491.	Desulfobacterium autotrophicum HRM2
492.	Desulfobacula toluolica Tol2
493.	Desulfobulbus propionicus DSM 2032
494.	Desulfocapsa sulfexigens DSM 10523
495.	Desulfohalobium retbaense DSM 5692
496.	Desulfomicrobium baculatum DSM 4028

497.	Desulfomonile tiedjei DSM 6799
498.	Desulfosporosinus acidiphilus SJ4
499.	Desulfosporosinus meridiei DSM 13257
500.	Desulfosporosinus orientis DSM 765
501.	Desulfotalea psychrophila LSv54
502.	Desulfotomaculum acetoxidans DSM 771
503.	Desulfotomaculum carboxydivorans CO-1-SRB
504.	Desulfotomaculum kuznetsovii DSM 6115
505.	Desulfotomaculum reducens MI-1
506.	Desulfotomaculum ruminis DSM 2154
507.	Desulfovibrio aespoeensis Aspo-2
508.	Desulfovibrio africanus str. Walvis Bay
509.	Desulfovibrio alaskensis G20
510.	Desulfovibrio desulfuricans ND132
511.	Desulfovibrio desulfuricans ssp. desulfuricans str. ATCC 27774
512.	Desulfovibrio hydrothermalis AM13 = DSM 14728
513.	Desulfovibrio magneticus RS-1
514.	Desulfovibrio piezophilus str. nov C1TLV30
515.	Desulfovibrio salexigens DSM 2638
516.	Desulfovibrio vulgaris RCH1
517.	Desulfovibrio vulgaris str. Miyazaki F
518.	Desulfovibrio vulgaris subsp. vulgaris DP4.
519.	Desulfovibrio vulgaris ssp. vulgaris str. Hildenborough
520.	Desulfurispirillum indicum S5
521.	Desulfurivibrio alkaliphilus AHT2
522.	Desulfurobacterium thermolithotrophum DSM 11699
523.	Desulfurococcus fermentans DSM 16532
524.	Desulfurococcus kamchatkensis 1221n
525.	Desulfurococcus mucosus DSM 2162
526.	Dichelobacter nodosus VCS1703A
527.	Dickeya dadantii 3937
528.	Dickeya dadantii Ech586

529.	Dickeya dadantii Ech703
530.	Dickeya zeae Ech1591
531.	Dictyoglomus thermophilum H-6-12
532.	Dictyoglomus turgidum DSM 6724
533.	Dinoroseobacter shibae DFL 12
534.	Dyadobacter fermentans DSM 18053
535.	Echinicola vietnamensis DSM 17526
536.	Edwardsiella ictaluri 93-146
537.	Edwardsiella tarda C07-087
538.	Edwardsiella tarda EIB202
539.	Edwardsiella tarda FL6-60
540.	Eggerthella lenta DSM 2243
541.	Eggerthella sp. YY7918
542.	Ehrlichia canis str. Jake
543.	Ehrlichia chaffeensis str. Arkansas
544.	Ehrlichia ruminantium str. Gardel
545.	Ehrlichia ruminantium str. Welgevonden Montpellier
546.	Ehrlichia ruminantium str. Welgevonden
547.	Elusimicrobium minutum Pei191
548.	Emticicia oligotrophica DSM 17448
549.	Enterobacter aerogenes EA1509E
550.	Enterobacter aerogenes KCTC 2190
551.	Enterobacter asburiae LF7a
552.	Enterobacter cloacae EcWSU1
553.	Enterobacter cloacae SCF1
554.	Enterobacter cloacae subsp. cloacae ATCC 13047
555.	Enterobacter cloacae ssp. cloacae ENHKU01
556.	Enterobacter cloacae ssp. dissolvens SDM
557.	Enterobacter sakazakii ATCC BAA-894
558.	Enterobacter sp. 638
559.	Enterobacteriaceae bacterium str.FGI 57
560.	Enterococcus faecalis 62

561.	Enterococcus faecalis D32
562.	Enterococcus faecalis OG1RF
563.	Enterococcus faecalis V583
564.	Enterococcus faecalis str. Symbioflor 1
565.	Enterococcus faecium Aus0004
566.	Enterococcus faecium DO
567.	Enterococcus faecium NRRL B-2354
568.	Enterococcus hirae ATCC 9790
569.	Eremothecium cymbalariae DBVPG 7215
570.	Erwinia amylovora
571.	Erwinia amylovora ATCC 49946
572.	Erwinia billingiae Eb661
573.	Erwinia carotovora ssp. atroseptica SCRI1043
574.	Erwinia pyrifoliae DSM 12163
575.	Erwinia pyrifoliae Ep1/96
576.	Erwinia sp. Ejp617
577.	Erwinia tasmaniensis
578.	Erysipelothrix rhusiopathiae str. Fujisawa
579.	Erythrobacter litoralis HTCC2594
580.	Escherichia blattae DSM 4481
581.	Escherichia coli 536
582.	Escherichia coli 55989
583.	Escherichia coli ABU 83972
584.	Escherichia coli APEC O1
585.	Escherichia coli APEC O78
586.	Escherichia coli ATCC 8739
587.	Escherichia coli B str. REL606
588.	Escherichia coli BL21DE3 Daejeon
589.	Escherichia coli BL21DE3 Wien
590.	Escherichia coli BL21-GoldDE3pLysS AG
591.	Escherichia coli BW2952
592.	Escherichia coli CFT073

593.	Escherichia coli DH1
594.	Escherichia coli DH10B
595.	Escherichia coli E24377A
596.	Escherichia coli ED1a
597.	Escherichia coli HS
598.	Escherichia coli IAI1
599.	Escherichia coli IAI39
600.	Escherichia coli IHE3034
601.	Escherichia coli KO11FL
602.	Escherichia coli KO11FL
603.	Escherichia coli LF82
604.	Escherichia coli NA114
605.	Escherichia coli O103:H2 str. 12009
606.	Escherichia coli O104:H4 str. 2009EL-2050
607.	Escherichia coli O104:H4 str. 2009EL-2071
608.	Escherichia coli O104:H4 str. 2011C-3493
609.	Escherichia coli O111:H- str. 11128
610.	Escherichia coli O127:H6 str. E2348/69
611.	Escherichia coli O157:H7j
612.	Escherichia coli O157:H7
613.	Escherichia coli O26:H11 str. 11368
614.	Escherichia coli O55:H7 str. CB9615
615.	Escherichia coli O55:H7 str. RM12579
616.	Escherichia coli O7:K1 str. CE10
617.	Escherichia coli P12b
618.	Escherichia coli S88
619.	Escherichia coli SE11
620.	Escherichia coli SE15
621.	Escherichia coli SMS-3-5
622.	Escherichia coli UM146
623.	Escherichia coli UMN026
624.	Escherichia coli UMNK88

625.	Escherichia coli UTI89
626.	Escherichia coli W
627.	Escherichia coli W
628.	Escherichia coli W3110
629.	Escherichia coli Xuzhou21
630.	Escherichia coli str. clone D i14
631.	Escherichia coli str. clone D i2
632.	Escherichia coli str. K-12 sstr. MG1655
633.	Escherichia fergusonii ATCC 35469
634.	Ethanoligenens harbinense YUAN-3
635.	Eubacterium eligens ATCC 27750
636.	Eubacterium limosum KIST612
637.	Eubacterium rectale ATCC 33656
638.	Exiguobacterium antarcticum B7
639.	Exiguobacterium sibiricum 255-15
640.	Exiguobacterium sp. AT1b
641.	Ferrimonas balearica DSM 9799
642.	Ferroglobus placidus DSM 10642
643.	Fervidicoccus fontis Kam940
644.	Fervidobacterium nodosum Rt17-B1
645.	Fervidobacterium pennivorans DSM 9078
646.	Fibrella aestuarina BUZ 2
647.	Fibrobacter succinogenes ssp. succinogenes S85
648.	Fibrobacter succinogenes ssp. succinogenes S85
649.	Filifactor alocis ATCC 35896
650.	Finegoldia magna ATCC 29328
651.	Flavobacteriaceae bacterium 3519-10
652.	Flavobacteriales bacterium HTCC2170
653.	Flavobacterium branchiophilum FL-15
654.	Flavobacterium columnare ATCC 49512
655.	Flavobacterium indicum GPTSA100-9
656.	Flavobacterium johnsoniae UW101

657.	Flavobacterium psychrophilum JIP02/86
658.	Flexibacter litoralis DSM 6794
659.	Flexistipes sinusarabici DSM 4947
660.	Fluviicola taffensis DSM 16823
661.	Francisella cf. novicida 3523
662.	Francisella cf. novicida Fx1
663.	Francisella noatunensis ssp. orientalis str. Toba 04
664.	Francisella philomiragia ssp. philomiragia ATCC 25017
665.	Francisella sp. TX077308
666.	Francisella tularensis TI0902
667.	Francisella tularensis TIGB03
668.	Francisella tularensis ssp. holarctica F92
669.	Francisella tularensis ssp. holarctica FSC200
670.	Francisella tularensis ssp. holarctica FTNF002-00
671.	Francisella tularensis ssp. holarctica LVS
672.	Francisella tularensis ssp. holarctica OSU18
673.	Francisella tularensis ssp. mediasiatica FSC147
674.	Francisella tularensis ssp. novicida U112
675.	Francisella tularensis ssp. tularensis FSC 198
676.	Francisella tularensis ssp. tularensis NE061598
677.	Francisella tularensis ssp. tularensis Schu 4
678.	Francisella tularensis ssp. tularensis WY96-3418
679.	Frankia alni ACN14a
680.	Frankia sp. CcI3
681.	Frankia sp. EAN1pec
682.	Frankia sp. EuI1c
683.	Frankia symbiont of Datisca glomerata
684.	Frateuria aurantia DSM 6220
685.	Fusobacterium nucleatum ATCC 25586
686.	Gallibacterium anatis UMN179
687.	Gallionella capsiferriformans ES-2
688.	Gamma proteobacterium HdN1

689.	Gardnerella vaginalis 409-05
690.	Gardnerella vaginalis ATCC 14019
691.	Gardnerella vaginalis HMP9231
692.	Geitlerinema sp. PCC 7407
693.	Gemmatimonas aurantiaca T-27
694.	Geobacillus kaustophilus HTA426
695.	Geobacillus sp. C56-T3
696.	Geobacillus sp. GHH01
697.	Geobacillus sp. WCH70
698.	Geobacillus sp. Y4.1MC1
699.	Geobacillus sp. Y412MC52
700.	Geobacillus sp. Y412MC61
701.	Geobacillus thermodenitrificans NG80-2
702.	Geobacillus thermoglucosidasius C56-YS93
703.	Geobacillus thermoleovorans CCB_US3_UF5
704.	Geobacter bemidjiensis Bem
705.	Geobacter lovleyi SZ
706.	Geobacter metallireducens GS-15
707.	Geobacter sp. FRC-32
708.	Geobacter sp. M18
709.	Geobacter sp. M21
710.	Geobacter sulfurreducens KN400
711.	Geobacter sulfurreducens
712.	Geobacter uraniumreducens Rf4
713.	Geodermatophilus obscurus DSM 43160
714.	Glaciecola nitratireducens FR1064
715.	Glaciecola sp. 4H-3-7+YE-5
716.	Gloeobacter violaceus
717.	Gloeocapsa sp. PCC 7428
718.	Gluconacetobacter diazotrophicus PAl 5
719.	Gluconacetobacter diazotrophicus PAl 5 ATCC 49037
720.	Gluconacetobacter xylinus NBRC 3288

721.	Gluconobacter oxydans 621H
722.	Gluconobacter oxydans H24
723.	Gordonia bronchialis DSM 43247
724.	Gordonia polyisoprenivorans VH2
725.	Gordonia sp. KTR9
726.	Gramella forsetii KT0803
727.	Granulobacter bethesdensis CGDNIH1
728.	Granulicella mallensis MP5ACTX8
729.	Haemophilus ducreyi 35000HP
730.	Haemophilus influenzae 10810
731.	Haemophilus influenzae 86-028NP
732.	Haemophilus influenzae F3031
733.	Haemophilus influenzae F3047
734.	Haemophilus influenzae PittEE
735.	Haemophilus influenzae PittGG
736.	Haemophilus influenzae R2846
737.	Haemophilus influenzae R2866
738.	Haemophilus influenzae Rd
739.	Haemophilus parainfluenzae T3T1
740.	Haemophilus parasuis SH0165
741.	Haemophilus somnus 129PT
742.	Haemophilus somnus 2336
743.	Hahella chejuensis KCTC 2396
744.	Halalkalicoccus jeotgali B3
745.	Halanaerobium hydrogeniformans
746.	Halanaerobium praevalens DSM 2228
747.	Haliangium ochraceum DSM 14365
748.	Halobacillus halophilus DSM 2266
749.	Halobacterium salinarum R1
750.	Halobacterium sp. NRC-1
751.	Haloferax mediterranei ATCC 33500
752.	Haloferax volcanii DS2

753.	Halomicrobium mukohataei DSM 12286
754.	Halomonas elongata DSM 2581
755.	Halophilic archaeon DL31
756.	Halopiger xanaduensis SH-6
757.	Haloquadratum walsbyi
758.	Haloquadratum walsbyi C23
759.	Halorhabdus utahensis DSM 12940
760.	Halorhodospira halophila SL1
761.	Halorubrum lacusprofundi ATCC 49239
762.	Haloterrigena turkmenica DSM 5511
763.	Halothece sp. PCC 7418
764.	Halothermothrix orenii H 168
765.	Halothiobacillus neapolitanus c2
766.	Halovivax ruber XH-70
767.	Helicobacter acinonychis str. Sheeba
768.	Helicobacter bizzozeronii CIII-1
769.	Helicobacter cetorum MIT 00-7128
770.	Helicobacter cetorum MIT 99-5656
771.	Helicobacter cinaedi ATCC BAA-847
772.	Helicobacter cinaedi PAGU611
773.	Helicobacter felis ATCC 49179
774.	Helicobacter hepaticus ATCC 51449
775.	Helicobacter mustelae 12198
776.	Helicobacter pylori 2017
777.	Helicobacter pylori 2018
778.	Helicobacter pylori, strain 26695
779.	Helicobacter pylori 26695
780.	Helicobacter pylori 35A
781.	Helicobacter pylori 51
782.	Helicobacter pylori 52
783.	Helicobacter pylori 83
784.	Helicobacter pylori 908

785.	Helicobacter pylori Aklavik117
786.	Helicobacter pylori Aklavik86
787.	Helicobacter pylori B38
788.	Helicobacter pylori B8
789.	Helicobacter pylori Cuz20
790.	Helicobacter pylori ELS37
791.	Helicobacter pylori F16
792.	Helicobacter pylori F30
793.	Helicobacter pylori F32
794.	Helicobacter pylori F57
795.	Helicobacter pylori G27
796.	Helicobacter pylori Gambia94/24
797.	Helicobacter pylori HPAG1
798.	Helicobacter pylori HUP-B14
799.	Helicobacter pylori India7
800.	Helicobacter pylori, strain J99
801.	Helicobacter pylori Lithuania75
802.	Helicobacter pylori OK113
803.	Helicobacter pylori P12
804.	Helicobacter pylori PeCan18
805.	Helicobacter pylori PeCan4
806.	Helicobacter pylori Puno120
807.	Helicobacter pylori Puno135
808.	Helicobacter pylori Rif1
809.	Helicobacter pylori Rif2
810.	Helicobacter pylori SJM180
811.	Helicobacter pylori SNT49
812.	Helicobacter pylori Sat464
813.	Helicobacter pylori Shi112
814.	Helicobacter pylori Shi169
815.	Helicobacter pylori Shi417
816.	Helicobacter pylori Shi470

817.	Helicobacter pylori SouthAfrica7
818.	Helicobacter pylori XZ274
819.	Helicobacter pylori v225d
820.	Heliobacterium modesticaldum Ice1
821.	Herbaspirillum seropedicae SmR1
822.	Herpetosiphon aurantiacus DSM 785
823.	Hippea maritima DSM 10411
824.	Hirschia baltica ATCC 49814
825.	Hydrogenobacter thermophilus TK-6
826.	Hydrogenobacter thermophilus TK-6 DSM 6534
827.	Hydrogenobaculum sp. 3684
828.	Hydrogenobaculum sp. HO
829.	Hydrogenobaculum sp. SHO
830.	Hydrogenobaculum sp. SN
831.	Hydrogenobaculum sp. Y04AAS1
832.	Hyperthermus butylicus DSM 5456
833.	Hyphomicrobium denitrificans ATCC 51888
834.	Hyphomicrobium sp. MC1
835.	Hyphomonas neptunium ATCC 15444
836.	Idiomarina loihiensis L2TR
837.	Ignavibacterium album JCM 16511
838.	Ignicoccus hospitalis KIN4/I
839.	Ignisphaera aggregans DSM 17230
840.	Ilyobacter polytropus DSM 2926
841.	Intrasporangium calvum DSM 43043
842.	Isoptericola variabilis 225
843.	Isosphaera pallida ATCC 43644
844.	Jannaschia sp. CCS1
845.	Janthinobacterium sp. Marseille
846.	Jonesia denitrificans DSM 20603
847.	Ketogulonicigenium vulgare Y25
848.	Ketogulonigenium vulgarum WSH-001

849.	Kineococcus radiotolerans SRS30216
850.	Klebsiella oxytoca E718
851.	Klebsiella pneumoniae 342
852.	Klebsiella pneumoniae KCTC 2242
853.	Klebsiella pneumoniae NTUH-K2044
854.	Klebsiella pneumoniae ssp. pneumoniae 1084
855.	Klebsiella pneumoniae ssp. pneumoniae HS11286
856.	Klebsiella pneumoniae subsp. pneumoniae HS11286
857.	Klebsiella variicola At-22
858.	Kluyveromyces thermotolerans st. CBS 6340
859.	Kocuria rhizophila DC2201
860.	Kribbella flavida DSM 17836
861.	Krokinobacter sp. 4H-3-7-5
862.	Kyrpidia tusciae DSM 2912
863.	Kytococcus sedentarius DSM 20547
864.	Lacinutrix sp. 5H-3-7-4
865.	Lactobacillus acidophilus 30SC
866.	Lactobacillus acidophilus NCFM
867.	Lactobacillus amylovorus GRL 1112
868.	Lactobacillus amylovorus GRL1118
869.	Lactobacillus brevis ATCC 367
870.	Lactobacillus buchneri CD034
871.	Lactobacillus buchneri NRRL B-30929
872.	Lactobacillus casei
873.	Lactobacillus casei ATCC 334
874.	Lactobacillus casei BD-II
875.	Lactobacillus casei LC2W
876.	Lactobacillus casei W56
877.	Lactobacillus casei str. Zhang
878.	Lactobacillus crispatus ST1
879.	Lactobacillus delbrueckii ssp. bulgaricus 2038
880.	Lactobacillus delbrueckii ssp. bulgaricus ATCC BAA-365

881.	Lactobacillus delbrueckii ssp. bulgaricus ND02
882.	Lactobacillus delbrueckii ssp. bulgaricus
883.	Lactobacillus fermentum CECT 5716
884.	Lactobacillus fermentum IFO 3956
885.	Lactobacillus gasseri ATCC 33323
886.	Lactobacillus helveticus DPC 4571
887.	Lactobacillus helveticus H10
888.	Lactobacillus helveticus R0052
889.	Lactobacillus johnsonii DPC 6026
890.	Lactobacillus johnsonii FI9785
891.	Lactobacillus johnsonii NCC 533
892.	Lactobacillus kefiranofaciens ZW3
893.	Lactobacillus plantarum JDM1
894.	Lactobacillus plantarum WCFS1
895.	Lactobacillus plantarum ZJ316
896.	Lactobacillus plantarum subsp. plantarum ST-III
897.	Lactobacillus reuteri DSM 20016
898.	Lactobacillus reuteri F275 kitasano
899.	Lactobacillus reuteri SD2112
900.	Lactobacillus rhamnosus ATCC 8530
901.	Lactobacillus rhamnosus GG
902.	Lactobacillus rhamnosus GG
903.	Lactobacillus rhamnosus Lc 705
904.	Lactobacillus ruminis ATCC 27782
905.	Lactobacillus sakei ssp. sakei 23K
906.	Lactobacillus salivarius CECT 5713
907.	Lactobacillus salivarius subsp. salivarius UCC118.
908.	Lactobacillus sanfranciscensis TMW 1.1304
909.	Lactococcus garvieae ATCC 49156
910.	Lactococcus garvieae Lg2
911.	Lactococcus lactis ssp. cremoris A76
912.	Lactococcus lactis ssp. cremoris MG1363

913.	Lactococcus lactis ssp. cremoris NZ9000
914.	Lactococcus lactis ssp. cremoris SK11
915.	Lactococcus lactis ssp. cremoris UC509.9
916.	Lactococcus lactis ssp. lactis CV56
917.	Lactococcus lactis IL1403
918.	Lactococcus lactis ssp. lactis IO-1
919.	Lactococcus lactis ssp. lactis KF147
920.	Laribacter hongkongensis HLHK9
921.	Lawsonia intracellularis N343
922.	Lawsonia intracellularis PHE/MN1-00.
923.	Leadbetterella byssophila DSM 17132
924.	Legionella longbeachae NSW150
925.	Legionella pneumophila 2300/99 Alcoy
926.	Legionella pneumophila str. Corby
927.	Legionella pneumophila str. Lens
928.	Legionella pneumophila str. Paris
929.	Legionella pneumophila ssp. pneumophila
930.	Legionella pneumophila subsp. pneumophila
931.	Legionella pneumophila ssp. pneumophila ATCC 43290
932.	Legionella pneumophila ssp. pneumophila str. Philadelphia 1
933.	Legionella pneumophila ssp. pneumophila str. Philadelphia 1
934.	Leifsonia xyli ssp. xyli str. CTCB07
935.	Leptolyngbya sp. PCC 7376
936.	Leptospira interrogans serovar Lai str. IPAV
937.	Leptospira interrogans serovar Lai str. IPAV
938.	Leptospirillum ferriphilum ML-04
939.	Leptospirillum ferrooxidans C2-3
940.	Leptothrix cholodnii SP-6
941.	Leptotrichia buccalis DSM 1135
942.	Leuconostoc carnosum JB16
943.	Leuconostoc citreum KM20
944.	Leuconostoc gasicomitatum LMG 18811

945.	Leuconostoc gelidum JB7
946.	Leuconostoc kimchii IMSNU11154
947.	Leuconostoc mesenteroides ssp. mesenteroides ATCC 8293
948.	Leuconostoc mesenteroides ssp. mesenteroides J18
949.	Leuconostoc sp. C2
950.	Liberibacter crescens BT-1
951.	Listeria innocua Clip11262
952.	Listeria ivanovii ssp. ivanovii PAM 55
953.	Listeria monocytogenes 07PF0776
954.	Listeria monocytogenes 08-5578
955.	Listeria monocytogenes 08-5923
956.	Listeria monocytogenes 10403S
957.	Listeria monocytogenes ATCC 19117
958.	Listeria monocytogenes Clip81459
959.	Listeria monocytogenes FSL R2-561
960.	Listeria monocytogenes Finland 1998
961.	Listeria monocytogenes HCC23
962.	Listeria monocytogenes J0161
963.	Listeria monocytogenes L312
964.	Listeria monocytogenes L99
965.	Listeria monocytogenes La111
966.	Listeria monocytogenes M7
967.	Listeria monocytogenes N53-1
968.	Listeria monocytogenes SLCC2372
969.	Listeria monocytogenes SLCC2376
970.	Listeria monocytogenes SLCC2378
971.	Listeria monocytogenes SLCC2479
972.	Listeria monocytogenes SLCC2540
973.	Listeria monocytogenes SLCC2755
974.	Listeria monocytogenes SLCC5850
975.	Listeria monocytogenes SLCC7179
976.	Listeria monocytogenes st. 4b str. LL195

977.	Listeria monocytogenes st. 7 str. SLCC2482
978.	Listeria monocytogenes str. 4b F2365
979.	Listeria monocytogenes EGD
980.	Listeria seeligeri serovar 1/2b str. SLCC3954
981.	Listeria welshimeri sv. 6b str. SLCC5334
982.	Lysinibacillus sphaericus C3-41
983.	Macrococcus caseolyticus JCSC5402
984.	Magnetococcus marinus MC-1
985.	Magnetospirillum magneticum AMB-1
986.	Mahella australiensis 50-1 BON
987.	Mannheimia succiniciproducens MBEL55E
988.	Maricaulis maris MCS10
989.	Marinithermus hydrothermalis DSM 14884
990.	Marinitoga piezophila KA3
991.	Marinobacter adhaerens HP15
992.	Marinobacter aquaeolei VT8.
993.	Marinobacter hydrocarbonoclasticus ATCC 49840
994.	Marinobacter sp. BSs20148
995.	Marinomonas mediterranea MMB-1
996.	Marinomonas posidonica IVIA-Po-181
997.	Marinomonas sp. MWYL1
998.	Marivirga tractuosa DSM 4126
999.	Megasphaera elsdenii DSM 20460
1000.	Meiothermus ruber DSM 1279
1001.	Meiothermus silvanus DSM 9946
1002.	Melioribacter roseus P3M
1003.	Melissococcus plutonius ATCC 35311
1004.	Melissococcus plutonius DAT561 1
1005.	Mesoplasma florum L1
1006.	Mesorhizobium australicum WSM2073
1007.	Mesorhizobium ciceri biovar biserrulae WSM1271
1008.	Mesorhizobium loti

1009.	Mesorhizobium opportunistum WSM2075
1010.	Mesorhizobium sp. BNC1.
1011.	Mesotoga prima MesG1.Ag.4.2
1012.	Metallosphaera cuprina Ar-4
1013.	Metallosphaera sedula DSM 5348
1014.	Methanobacterium sp. AL-21
1015.	Methanobacterium sp. SWAN-1
1016.	Methanobacterium thermoautotrophicum
1017.	Methanobrevibacter ruminantium M1
1018.	Methanobrevibacter smithii ATCC 35061
1019.	Methanocaldococcus fervens AG86
1020.	Methanocaldococcus infernus ME
1021.	Methanocaldococcus sp. FS406-22
1022.	Methanocaldococcus vulcanius M7
1023.	Methanocella arvoryzae MRE50
1024.	Methanocella conradii HZ254
1025.	Methanocella paludicola SANAE
1026.	Methanococcoides burtonii DSM 6242
1027.	Methanococcus aeolicus Nankai-3
1028.	Methanococcus jannaschii
1029.	Methanococcus maripaludis
1030.	Methanococcus maripaludis C5
1031.	Methanococcus maripaludis C6
1032.	Methanococcus maripaludis C7
1033.	Methanococcus maripaludis XI
1034.	Methanococcus vannielii SB
1035.	Methanococcus voltae A3
1036.	Methanocorpusculum labreanum Z
1037.	Methanoculleus bourgensis MS2
1038.	Methanoculleus marisnigri JR1
1039.	Methanohalobium evestigatum Z-7303
1040.	Methanohalophilus mahii DSM 5219

1041.	Methanolobus psychrophilus R15
1042.	Methanomethylovorans hollandica DSM 15978
1043.	Methanoplanus petrolearius DSM 11571
1044.	Methanopyrus kandleri AV19
1045.	Methanoregula formicicum SMSP
1046.	Methanosaeta concilii GP-6
1047.	Methanosaeta harundinacea 6Ac
1048.	Methanosaeta thermophila PT Methanothrix thermophila PT
1049.	Methanosalsum zhilinae DSM 4017
1050.	Methanosarcina acetivorans str. C2A
1051.	Methanosarcina barkeri str. fusaro
1052.	Methanosarcina mazei Tuc01
1053.	Methanosarcina mazei Goe1
1054.	Methanosphaera stadtmanae DSM 3091
1055.	Methanospirillum hungatei JF-1
1056.	Methanothermobacter marburgensis str. Marburg
1057.	Methanothermococcus okinawensis IH1
1058.	Methanothermus fervidus DSM 2088
1059.	Methanotorris igneus Kol 5
1060.	Methylacidiphilum infernorum V4
1061.	Methylibium petroleiphilum PM1
1062.	Methylobacillus flagellatus KT
1063.	Methylobacterium chloromethanicum CM4
1064.	Methylobacterium extorquens AM1
1065.	Methylobacterium extorquens DM4
1066.	Methylobacterium extorquens PA1
	Methylobacterium nodulans ORS 2060
1067.	Methylobacterium populi BJ001
1068.	Methylobacterium radiotolerans JCM 2831
1069.	Methylobacterium sp. 4-46
1070.	Methylocella silvestris BL2
1071.	Methylococcus capsulatus str. Bath

1072.	Methylocystis sp. SC2
1073.	Methylomicrobium alcaliphilum
1074.	Methylomonas methanica MC09
1075.	Methylophaga sp. JAM1
1076.	Methylophaga sp. JAM7
1077.	Methylotenera mobilis JLW8
1078.	Methylotenera sp. 301
1079.	Methylovorus sp. MP688
1080.	Methylovorus sp. SIP3-4
1081.	Micavibrio aeruginosavorus ARL-13
1082.	Micavibrio aeruginosavorus EPB
1083.	Microbacterium testaceum StLB037
1084.	Micrococcus luteus NCTC 2665
1085.	Microcoleus sp. PCC 7113
1086.	Microcystis aeruginosa NIES-843
1087.	Microlunatus phosphovorus NM-1
1088.	Micromonospora aurantiaca ATCC 27029
1089.	Micromonospora sp. L5
1090.	Mobiluncus curtisii ATCC 43063
1091.	Modestobacter marinus
1092.	Moorella thermoacetica ATCC 39073
1093.	Moraxella catarrhalis RH4
1094.	Morganella morganii ssp. morganii KT
1095.	Muricauda ruestringensis DSM 13258
1096.	Mycobacterium africanum GM041182
1097.	Mycobacterium avium 104
1098.	Mycobacterium avium paratuberculosis
1099.	Mycobacterium bovis BCG str. Korea 1168P
1100.	Mycobacterium bovis BCG str. Mexico
1101.	Mycobacterium bovis BCG str. Pasteur 1173P2
1102.	Mycobacterium bovis BCG str. Tokyo 172
1103.	Mycobacterium bovis ssp. bovis AF2122/97

1104.	Mycobacterium canettii CIPT 140010059
1105.	Mycobacterium canettii CIPT 140060008
1106.	Mycobacterium canettii CIPT 140070008
1107.	Mycobacterium canettii CIPT 140070010
1108.	Mycobacterium canettii CIPT 140070017
1109.	Mycobacterium chubuense NBB4
1110.	Mycobacterium gilvum PYR-GCK
1111.	Mycobacterium indicus pranii MTCC 9506
1112.	Mycobacterium intracellulare ATCC 13950
1113.	Mycobacterium intracellulare MOTT-02
1114.	Mycobacterium intracellulare MOTT-64
1115.	Mycobacterium leprae Br4923
1116.	Mycobacterium leprae TN
1117.	Mycobacterium liflandii 128FXT
1118.	Mycobacterium marinum M
1119.	Mycobacterium massiliense str. GO 06
1120.	Mycobacterium rhodesiae NBB3
1121.	Mycobacterium smegmatis JS623
1122.	Mycobacterium smegmatis str. MC2 155 v2
1123.	Mycobacterium smegmatis str. MC2 155
1124.	Mycobacterium sp. JDM601
1125.	Mycobacterium sp. JLS
1126.	Mycobacterium sp. KMS.
1127.	Mycobacterium sp. MCS.
1128.	Mycobacterium sp. MOTT36Y
1129.	Mycobacterium sp. Spyr1
1130.	Mycobacterium tuberculosis CCDC5079
1131.	Mycobacterium tuberculosis CCDC5180
1132.	Mycobacterium tuberculosis CDC1551
1133.	Mycobacterium tuberculosis CTRI-2
1134.	Mycobacterium tuberculosis F11
1135.	Mycobacterium tuberculosis H37Ra

1136.	Mycobacterium tuberculosis H37Rv
1137.	Mycobacterium tuberculosis H37Rv
1138.	Mycobacterium tuberculosis KZN 1435
1139.	Mycobacterium tuberculosis KZN 4207
1140.	Mycobacterium tuberculosis KZN 605
1141.	Mycobacterium tuberculosis RGTB327
1142.	Mycobacterium tuberculosis RGTB423
1143.	Mycobacterium tuberculosis UT205
1144.	Mycobacterium ulcerans Agy99
1145.	Mycobacterium vanbaalenii PYR-1
1146.	Mycoplasma agalactiae
1147.	Mycoplasma agalactiae PG2
1148.	Mycoplasma arthritidis 158L3-1
1149.	Mycoplasma bovis HB0801
1150.	Mycoplasma bovis Hubei-1
1151.	Mycoplasma bovis PG45
1152.	Mycoplasma capricolum ssp. capricolum ATCC 27343
1153.	Mycoplasma conjunctivae
1154.	Mycoplasma crocodyli MP145
1155.	Mycoplasma cynos C142
1156.	Mycoplasma fermentans JER
1157.	Mycoplasma fermentans M64
1158.	Mycoplasma gallisepticum CA06_2006.052-5-2P
1159.	Mycoplasma gallisepticum NC06_2006.080-5-2P
1160.	Mycoplasma gallisepticum NC08_2008.031-4-3P
1161.	Mycoplasma gallisepticum NC95_13295-2-2P
1162.	Mycoplasma gallisepticum NC96_1596-4-2P
1163.	Mycoplasma gallisepticum NY01_2001.047-5-1P
1164.	Mycoplasma gallisepticum R
1165.	Mycoplasma gallisepticum VA94_7994-1-7P
1166.	Mycoplasma gallisepticum WI01_2001.043-13-2P
1167.	Mycoplasma gallisepticum str. F

1168.	Mycoplasma gallisepticum str. Rhigh
1169.	Mycoplasma genitalium G37
1170.	Mycoplasma genitalium M2288
1171.	Mycoplasma genitalium M2321
1172.	Mycoplasma genitalium M6282
1173.	Mycoplasma genitalium M6320
1174.	Mycoplasma haemocanis str. Illinois
1175.	Mycoplasma haemofelis Ohio2
1176.	Mycoplasma haemofelis str. Langford 1
1177.	Mycoplasma hominis ATCC 23114
1178.	Mycoplasma hyopneumoniae 168
1179.	Mycoplasma hyopneumoniae 232
1180.	Mycoplasma hyopneumoniae 7448
1181.	Mycoplasma hyopneumoniae J
1182.	Mycoplasma hyorhinis GDL-1
1183.	Mycoplasma hyorhinis HUB-1
1184.	Mycoplasma hyorhinis MCLD
1185.	Mycoplasma hyorhinis SK76
1186.	Mycoplasma leachii 99/014/6
1187.	Mycoplasma leachii PG50
1188.	Mycoplasma mobile 163K
1189.	Mycoplasma mycoides ssp. capri LC str. 95010
1190.	Mycoplasma mycoides
1191.	Mycoplasma penetrans
1192.	Mycoplasma pneumoniae 309
1193.	Mycoplasma pneumoniae FH
1194.	Mycoplasma pneumoniae M129
1195.	Mycoplasma pulmonis
1196.	Mycoplasma putrefaciens KS1
1197.	Mycoplasma suis KI_3806
1198.	Mycoplasma suis str. Illinois
1199.	Mycoplasma synoviae 53

1200.	Mycoplasma wenyonii str. Massachusetts
1201.	Myxococcus fulvus HW-1
1202.	Myxococcus stipitatus DSM 14675
1203.	Myxococcus xanthus DK 1622
1204.	NC10 bacterium Dutch sediment
1205.	Nakamurella multipartita DSM 44233
1206.	Nanoarchaeum equitans Kin4-M
1207.	Natranaerobius thermophilus JW/NM-WN-LF
1208.	Natrinema pellirubrum DSM 15624
1209.	Natrinema sp. J7-2
1210.	Natronobacterium gregoryi SP2
1211.	Natronococcus occultus SP4
1212.	Natronomonas moolapensis 8.8.11
1213.	Nautilia profundicola AmH
1214.	Neisseria gonorrhoeae FA 1090
1215.	Neisseria gonorrhoeae NCCP11945
1216.	Neisseria gonorrhoeae TCDC-NG08107
1217.	Neisseria lactamica ST-640
1218.	Neisseria meningitidis 053442
1219.	Neisseria meningitidis 8013
1220.	Neisseria meningitidis A/Z2491
1221.	Neisseria meningitidis FAM18
1222.	Neisseria meningitidis G2136
1223.	Neisseria meningitidis H44/76
1224.	Neisseria meningitidis M01-240149
1225.	Neisseria meningitidis M01-240355
1226.	Neisseria meningitidis M04-240196
1227.	Neisseria meningitidis NZ-05/33
1228.	Neisseria meningitidis WUE 2594
1229.	Neisseria meningitidis alpha14
1230.	Neisseria meningitidis alpha710
1231.	Neisseria meningitidis B/MC58

1232.	Neorickettsia risticii str. Illinois
1233.	Neorickettsia sennetsu str. Miyayama
1234.	Niastella koreensis GR20-10
1235.	Nitratifractor salsuginis DSM 16511
1236.	Nitratiruptor sp. SB155-2
1237.	Nitrobacter hamburgensis X14.
1238.	Nitrobacter winogradskyi Nb-255
1239.	Nitrosococcus halophilus Nc4
1240.	Nitrosococcus oceani ATCC 19707
1241.	Nitrosococcus watsoni C-113
1242.	Nitrosomonas europaea ATCC 19718
1243.	Nitrosomonas eutropha C71
1244.	Nitrosomonas sp. AL212
1245.	Nitrosomonas sp. Is79A3
1246.	Nitrosopumilus maritimus SCM1
1247.	Nitrosospira multiformis ATCC 25196
1248.	Nocardia brasiliensis ATCC 700358
1249.	Nocardia cyriacigeorgica GUH-2
1250.	Nocardia farcinica
1251.	Nocardioides sp. JS614.
1252.	Nocardiopsis alba ATCC BAA-2165
1253.	Nocardiopsis dassonvillei subsp. dassonvillei DSM 43111,
1254.	Nonlabens dokdonensis DSW-6
1255.	Nostoc punctiforme PCC 73102
1256.	Nostoc sp. PCC 7107
1257.	Nostoc sp. PCC 7120
1258.	Nostoc sp. PCC 7524
1259.	Novosphingobium aromaticivorans DSM 12444
1260.	Novosphingobium sp. PP1Y
1261.	Novosphingobium sp. PP1Y
1262.	Oceanimonas sp. GK1
1263.	Oceanithermus profundus DSM 14977

1264.	Oceanobacillus iheyensis
1265.	Odoribacter splanchnicus DSM 20712
1266.	Oenococcus oeni PSU-1
1267.	Oligotropha carboxidovorans OM4
1268.	Oligotropha carboxidovorans OM5 ATCC 49405
1269.	Oligotropha carboxidovorans OM5 DSM 1227
1270.	Olsenella uli DSM 7084
1271.	Opitutus terrae PB90-1
1272.	Orientia tsutsugamushi Boryong
1273.	Orientia tsutsugamushi str. Ikeda
1274.	Ornithobacterium rhinotracheale DSM 15997
1275.	Oscillatoria acuminata PCC 6304
1276.	Oscillatoria nigro-viridis PCC 7112
1277.	Oscillibacter valericigenes Sjm18-20
1278.	Owenweeksia hongkongensis DSM 17368
1279.	Paenibacillus mucilaginosus 3016
1280.	Paenibacillus mucilaginosus K02
1281.	Paenibacillus mucilaginosus KNP414
1282.	Paenibacillus polymyxa E681
1283.	Paenibacillus polymyxa M1
1284.	Paenibacillus polymyxa SC2
1285.	Paenibacillus sp. JDR-2
1286.	Paenibacillus sp. Y412MC10
1287.	Paenibacillus terrae HPL-003
1288.	Paludibacter propionicigenes WB4
1289.	Pantoea ananatis AJ13355
1290.	Pantoea ananatis LMG 20103
1291.	Pantoea ananatis LMG 5342
1292.	Pantoea ananatis PA13
1293.	Pantoea vagans C9-1
1294.	Parabacteroides distasonis ATCC 8503
1295.	Parachlamydia acanthamoebae UV7

1296.	Parachlamydia sp. UWE25
1297.	Parvibaculum lavamentivorans DS-1
1298.	Parvularcula bermudensis HTCC2503
1299.	Pasteurella multocida 36950
1300.	Pasteurella multocida PM70
1301.	Pasteurella multocida ssp. multocida str. 3480
1302.	Pasteurella multocida ssp. multocida str. HN06
1303.	Pectobacterium carotovorum ssp. carotovorum PC1
1304.	Pectobacterium carotovorum ssp. carotovorum PCC21
1305.	Pectobacterium sp. SCC3193
1306.	Pectobacterium wasabiae WPP163
1307.	Pediococcus claussenii ATCC BAA-344
1308.	Pediococcus pentosaceus ATCC 25745
1309.	Pedobacter heparinus DSM 2366
1310.	Pedobacter saltans DSM 12145
1311.	Pelagibacterium halotolerans B2
1312.	Pelobacter carbinolicus DSM 2380
1313.	Pelobacter propionicus DSM 2379.
1314.	Pelodictyon luteolum DSM 273
1315.	Pelodictyon phaeoclathratiforme BU-1
1316.	Pelotomaculum thermopropionicum SI
1317.	Penicillium chrysogenum Wisconsin 54-1255
1318.	Persephonella marina EX-H1
1319.	Petrotoga mobilis SJ95
1320.	Phaeobacter gallaeciensis 2.10
1321.	Phaeobacter gallaeciensis DSM 17395
1322.	Phenylobacterium zucineum HLK1
1323.	Photorhabdus asymbiotica
1324.	Photorhabdus luminescens ssp. laumondii TTO1
1325.	Phycisphaera mikurensis NBRC 102666
1326.	Picrophilus torridus DSM 9790
1327.	Pirellula sp.

1328.	Pirellula staleyi DSM 6068
1329.	Planctomyces brasiliensis DSM 5305
1330.	Planctomyces limnophilus DSM 3776
1331.	Pleurocapsa sp. PCC 7327
1332.	Polaromonas naphthalenivorans CJ2.
1333.	Polaromonas sp. JS666
1334.	Polymorphum gilvum SL003B-26A1
1335.	Polynucleobacter necessarius STIR1
1336.	Polynucleobacter sp. QLW-P1DMWA-1
1337.	Porphyromonas asaccharolytica DSM 20707
1338.	Porphyromonas gingivalis ATCC 33277
1339.	Porphyromonas gingivalis TDC60
1340.	Porphyromonas gingivalis W83
1341.	Prevotella dentalis DSM 3688
1342.	Prevotella dentalis DSM 3688
1343.	Prevotella denticola F0289
1344.	Prevotella ruminicola 23
1345.	Prochlorococcus marinus str. AS9601
1346.	Prochlorococcus marinus str. MIT 9215
1347.	Prochlorococcus marinus str. MIT 9301
1348.	Prochlorococcus marinus str. MIT 9303
1349.	Prochlorococcus marinus str. MIT 9312
1350.	Prochlorococcus marinus str. MIT 9313
1351.	Prochlorococcus marinus str. NATL1A
1352.	Prochlorococcus marinus str. NATL2A
1353.	Prochlorococcus marinus ssp. marinus str. CCMP1375
1354.	Prochlorococcus marinus ssp. pastoris str. CCMP1986
1355.	Propionibacterium acnes 266
1356.	Propionibacterium acnes 6609
1357.	Propionibacterium acnes ATCC 11828
1358.	Propionibacterium acnes C1
1359.	Propionibacterium acnes KPA171202

1360.	Propionibacterium acnes SK137
1361.	Propionibacterium acnes TypeIA2 P.acn17
1362.	Propionibacterium acnes TypeIA2 P.acn31
1363.	Propionibacterium acnes TypeIA2 P.acn33
1364.	Propionibacterium freudenreichii ssp. shermanii CIRM-BIA1
1365.	Propionibacterium propionicum F0230a
1366.	Prosthecochloris aestuarii DSM 271
1367.	Prosthecochloris vibrioformis DSM 265
1368.	Proteus mirabilis HI4320
1369.	Providencia stuartii MRSN 2154
1370.	Pseudanabaena sp. PCC 7367
1371.	Pseudoalteromonas atlantica T6c
1372.	Pseudogulbenkiania sp. NH8B
1373.	Pseudomonas aeruginosa DK2
1374.	Pseudomonas aeruginosa LESB58
1375.	Pseudomonas aeruginosa M18
1376.	Pseudomonas aeruginosa NCGM2.S1
1377.	Pseudomonas aeruginosa
1378.	Pseudomonas aeruginosa PA7
1379.	Pseudomonas aeruginosa UCBPP-PA14
1380.	Pseudomonas brassicacearum ssp. brassicacearum NFM421
1381.	Pseudomonas entomophila L48
1382.	Pseudomonas fluorescens A506
1383.	Pseudomonas fluorescens F113
1384.	Pseudomonas fluorescens Pf-5
1385.	Pseudomonas fluorescens Pf0-1
1386.	Pseudomonas fluorescens SBW25
1387.	Pseudomonas fulva 12-X
1388.	Pseudomonas mendocina NK-01
1389.	Pseudomonas mendocina ymp
1390.	Pseudomonas poae RE*1-1-14
1391.	Pseudomonas putida BIRD-1

1392.	Pseudomonas putida DOT-T1E
1393.	Pseudomonas putida F1
1394.	Pseudomonas putida GB-1
1395.	Pseudomonas putida HB3267
1396.	Pseudomonas putida KT2440
1397.	Pseudomonas putida ND6
1398.	Pseudomonas putida S16
1399.	Pseudomonas putida UW4
1400.	Pseudomonas putida W619
1401.	Pseudomonas stutzeri A1501
1402.	Pseudomonas stutzeri ATCC 17588 = LMG 11199
1403.	Pseudomonas stutzeri CCUG 29243
1404.	Pseudomonas stutzeri DSM 10701
1405.	Pseudomonas stutzeri DSM 4166
1406.	Pseudomonas stutzeri RCH2
1407.	Pseudomonas syringae pv. phaseolicola 1448A.
1408.	Pseudomonas syringae pv. syringae B728a
1409.	Pseudonocardia dioxanivorans CB1190
1410.	Pseudovibrio sp. FO-BEG1
1411.	Pseudoxanthomonas spadix BD-a59
1412.	Pseudoxanthomonas suwonensis 11-1
1413.	Psychrobacter arcticum 273-4
1414.	Psychrobacter cryohalolentis K5
1415.	Psychrobacter sp. PRwf-1
1416.	Psychroflexus torquis ATCC 700755
1417.	Psychromonas ingrahamii 37
1418.	Psychromonas sp. CNPT3
1419.	Pusillimonas sp. T7-7
1420.	Pyrobaculum aerophilum
1421.	Pyrobaculum arsenaticum DSM 13514
1422.	Pyrobaculum calidifontis JCM 11548
1423.	Pyrobaculum islandicum DSM 4184

1424.	Pyrobaculum oguniense TE7
1425.	Pyrobaculum sp. 1860
1426.	Pyrococcus abyssi
1427.	Pyrococcus furiosus COM1
1428.	Pyrococcus horikoshii OT3
1429.	Pyrococcus sp. NA2
1430.	Pyrococcus sp. ST04
1431.	Pyrococcus yayanosii CH1
1432.	Pyrolobus fumarii 1A
1433.	Rahnella aquatilis CIP 78.65 = ATCC 33071,
1434.	Rahnella aquatilis HX2
1435.	Rahnella sp. Y9602
1436.	Ralstonia solanacearum CFBP2957
1437.	Ralstonia solanacearum PSI07
1438.	Ralstonia solanacearum Po82
1439.	Ramlibacter tataouinensis TTB310
1440.	Renibacterium salmoninarum ATCC 33209
1441.	Rhizobium etli CFN 42.
1442.	Rhizobium etli CIAT 652
1443.	Rhizobium leguminosarum bv. trifolii WSM1325
1444.	Rhizobium leguminosarum bv. viciae 3841.
1445.	Rhizobium sp. NGR234
1446.	Rhizobium tropici CIAT 899
1447.	Rhodanobacter sp. 2APBS1
1448.	Rhodobacter capsulatus SB1003
1449.	Rhodococcus equi 103S
1450.	Rhodococcus erythropolis PR4
1451.	Rhodococcus jostii RHA1
1452.	Rhodococcus opacus B4
1453.	Rhodoferax ferrireducens DSM 15236.
1454.	Rhodomicrobium vannielii ATCC 17100
1455.	Rhodopseudomonas palustris CGA009

1456.	Rhodopseudomonas palustris BisA53
1457.	Rhodopseudomonas palustris BisB18
1458.	Rhodopseudomonas palustris BisB5
1459.	Rhodopseudomonas palustris DX-1
1460.	Rhodopseudomonas palustris HaA2
1461.	Rhodopseudomonas palustris TIE-1
1462.	Rhodospirillum centenum SW
1463.	Rhodospirillum photometricum DSM 122
1464.	Rhodospirillum rubrum ATCC 11170
1465.	Rhodospirillum rubrum F11
1466.	Rhodothermus marinus DSM 4252
1467.	Rhodothermus marinus DSM 4252
1468.	Rhodothermus marinus SG0.5JP17-172
1469.	Rickettsia africae ESF-5
1470.	Rickettsia akari str. Hartford
1471.	Rickettsia australis str. Cutlack
1472.	Rickettsia bellii OSU 85-389
1473.	Rickettsia bellii RML369-C
1474.	Rickettsia canadensis str. CA410
1475.	Rickettsia canadensis str. McKiel
1476.	Rickettsia conorii Malish 7
1477.	Rickettsia felis URRWXCal2
1478.	Rickettsia heilongjiangensis 054
1479.	Rickettsia japonica YH
1480.	Rickettsia massiliae MTU5
1481.	Rickettsia massiliae str. AZT80
1482.	Rickettsia montanensis str. OSU 85-930
1483.	Rickettsia parkeri str. Portsmouth
1484.	Rickettsia peacockii str. Rustic
1485.	Rickettsia philipii str. 364D
1486.	Rickettsia prowazekii Rp22
1487.	Rickettsia prowazekii str. Chernikova

1488.	Rickettsia prowazekii str. Dachau
1489.	Rickettsia prowazekii str. Katsinyian
1490.	Rickettsia prowazekii str. RpGvF24
1491.	Rickettsia prowazekii Madrid E
1492.	Rickettsia rhipicephali str. 3-7-female6-CWPP
1493.	Rickettsia rickettsii str. Sheila Smith
1494.	Rickettsia rickettsii str. Arizona
1495.	Rickettsia rickettsii str. Brazil
1496.	Rickettsia rickettsii str. Colombia
1497.	Rickettsia rickettsii str. Hauke
1498.	Rickettsia rickettsii str. Hino
1499.	Rickettsia rickettsii str. Hlp 2
1500.	Rickettsia rickettsii str. Iowa
1501.	Rickettsia slovaca 13-B
1502.	Rickettsia slovaca str. D-CWPP
1503.	Rickettsia typhi str. B9991CWPP
1504.	Rickettsia typhi str. TH1527
1505.	Rickettsia typhi str. wilmington
1506.	Riemerella anatipestifer DSM 15868
1507.	Riemerella anatipestifer DSM 15868/ATCC 11845
1508.	Riemerella anatipestifer RA-CH-1
1509.	Riemerella anatipestifer RA-CH-2
1510.	Riemerella anatipestifer RA-GD
1511.	Rivularia sp. PCC 7116
1512.	Robiginitalea biformata HTCC2501
1513.	Roseburia hominis A2-183
1514.	Roseiflexus castenholzii DSM 13941
1515.	Roseiflexus sp. RS-1
1516.	Roseobacter denitrificans OCh 114
1517.	Roseobacter litoralis Och 149
1518.	Rothia dentocariosa ATCC 17931
1519.	Rothia mucilaginosa DY-18

1520.	Rubrivivax gelatinosus IL144
1521.	Rubrobacter xylanophilus DSM 9941
1522.	Ruegeria pomeroyi DSS-3
1523.	Ruminococcus albus 7
1524.	Runella slithyformis DSM 19594
1525.	Saccharomonospora viridis DSM 43017
1526.	Saccharomyces cerevisiae mitochondrion
1527.	Saccharophagus degradans 2-40
1528.	Saccharopolyspora erythraea NRRL 2338
1529.	Saccharothrix espanaensis DSM 44229
1530.	Salinibacter ruber DSM 13855
1531.	Salinibacter ruber M8
1532.	Salinispora arenicola CNS-205
1533.	Salinispora tropica CNB-440
1534.	Salmonella bongori NCTC 12419
1535.	Salmonella enterica S.typhi CT18
1536.	Salmonella enterica ssp. enterica serovar Typhimurium str. ST4/74
1537.	Salmonella enterica ssp. arizonae sv 62:z4
1538.	Salmonella enterica ssp. enterica sv. Agona str. SL483
1539.	Salmonella enterica ssp. enterica sv. Choleraesuis str
1540.	Salmonella enterica ssp. enterica sv. Dublin str. CT_02021853
1541.	Salmonella enterica ssp. enterica sv. Enteritidis str. P125109
1542.	Salmonella enterica ssp. enterica sv. Gallinarum str. 287/91
1543.	Salmonella enterica ssp. enterica sv. Gallinarum/pullorum
1544.	Salmonella enterica ssp. enterica sv. Heidelberg str. B182
1545.	Salmonella enterica ssp. enterica serovar Heidelberg str. SL476,
1546.	Salmonella enterica ssp. enterica sv. Javiana
1547.	Salmonella enterica subsp. enterica serovar Newport str. SL254,
1548.	Salmonella enterica enterica sv Newport str. SL254
1549.	Salmonella enterica ssp. enterica sv. Paratyphi A str. AKU_12601
1550.	Salmonella enterica sp. enterica sv. Paratyphi A str. ATCC
1551.	Salmonella enterica ssp. enterica sv Paratyphi B str. SPB7

1552.	Salmonella enterica ssp. enterica sv. Paratyphi C strain
1553.	Salmonella enterica subsp. enterica serovar Schwarzengrund str. CVM19633
1554.	Salmonella enterica ssp. enterica ser. Typhi Ty2
1555.	Salmonella enterica subsp. enterica serovar Typhi str. P-stx-12
1556.	Salmonella enterica ssp. enterica sv. Typhimurium str. 14028S
1557.	Salmonella enterica subsp. enterica serovar Typhimurium str. 798
1558.	Salmonella enterica ssp. enterica sv. Typhimurium str. D23580
1559.	Salmonella enterica ssp. enterica sv. Typhimurium str. SL1344
1560.	Salmonella enterica subsp. enterica serovar Typhimurium str. T000240
1561.	Salmonella enterica subsp. enterica serovar Typhimurium str. UK-1
1562.	Salmonella typhimurium LT2
1563.	Sanguibacter keddieii DSM 10542
1564.	Saprospira grandis str. Lewin
1565.	Sebaldella termitidis ATCC 33386
1566.	Secondary endosymbiont of Heteropsylla cubana
1567.	Segniliparus rotundus DSM 44985
1568.	Selenomonas ruminantium subsp. lactilytica TAM6421
1569.	Selenomonas sputigena ATCC 35185
1570.	Serratia marcescens FGI94
1571.	Serratia marcescens WW4
1572.	Serratia plymuthica AS9
1573.	Serratia proteamaculans 568
1574.	Serratia sp. AS12
1575.	Serratia sp. AS13
1576.	Serratia symbiotica str. Cinara cedri
1577.	Shewanella amazonensis SB2B
1578.	Shewanella baltica BA175
1579.	Shewanella baltica OS117
1580.	Shewanella baltica OS155
1581.	Shewanella baltica OS185
1582.	Shewanella baltica OS195
1583.	Shewanella baltica OS223

1584.	Shewanella baltica OS678
1585.	Shewanella denitrificans OS217
1586.	Shewanella frigidimarina NCIMB 400
1587.	Shewanella halifaxensis HAW-EB4
1588.	Shewanella loihica PV-4
1589.	Shewanella oneidensis MR-1
1590.	Shewanella pealeana ATCC 700345
1591.	Shewanella piezotolerans WP3
1592.	Shewanella putrefaciens 200
1593.	Shewanella putrefaciens CN-32
1594.	Shewanella sediminis HAW-EB3
1595.	Shewanella sp. MR-4
1596.	Shewanella sp. MR-7
1597.	Shewanella sp. W3-18-1
1598.	Shewanella violacea DSS12
1599.	Shewanella woodyi ATCC 51908
1600.	Shigella boydii CDC 3083-94
1601.	Shigella boydii Sb227
1602.	Shigella dysenteriae Sd197.
1603.	Shigella flexneri 2002017
1604.	Shigella flexneri 2a st. 2457T
1605.	Shigella flexneri 2a st. 301
1606.	Shigella flexneri 5 str. 8401
1607.	Shigella sonnei 53G
1608.	Shigella sonnei Ss046.
1609.	Sideroxydans lithotrophicus ES-1
1610.	Silicibacter sp. TM1040.
1611.	Simiduia agarivorans SA1 = DSM 21679
1612.	Singulisphaera acidiphila DSM 18658
1613.	Sinorhizobium fredii USDA 257
1614.	Sinorhizobium medicae WSM419
1615.	Sinorhizobium meliloti 2011

1616.	Sinorhizobium meliloti BL225C
1617.	Sinorhizobium meliloti GR4
1618.	Sinorhizobium meliloti Rm41
1619.	Sinorhizobium meliloti SM11
1620.	Sinorhizobium meliloti 1021
1621.	Slackia heliotrinireducens DSM 20476
1622.	Sodalis glossinidius str. morsitans
1623.	Solibacillus silvestris StLB046
1624.	Solibacter usitatus Ellin6076
1625.	Solitalea canadensis DSM 3403
1626.	Sorangium cellulosum So ce 56
1627.	Sphaerochaeta pleomorpha str. Grapes
1628.	Sphingobacterium sp. 21
1629.	Sphingobium sp. SYK-6
1630.	Sphingomonas sp. MM-1
1631.	Sphingomonas wittichii RW1
1632.	Sphingopyxis alaskensis RB2256
1633.	Spirochaeta africana DSM 8902
1634.	Spirochaeta caldaria DSM 7334
1635.	Spirochaeta coccoides DSM 17374
1636.	Spirochaeta smaragdinae DSM 11293
1637.	Spirochaeta sp. Buddy
1638.	Spirochaeta thermophila DSM 6192
1639.	Spirochaeta thermophila DSM 6578
1640.	Spirosoma linguale DSM 74
1641.	Stackebrandtia nassauensis DSM 44728
1642.	Stanieria cyanosphaera PCC 7437
1643.	Staphylococcus aureus 04-02981
1644.	Staphylococcus aureus 08BA02176
1645.	Staphylococcus aureus RF122
1646.	Staphylococcus aureus Mu50
1647.	Staphylococcus aureus N315

1648.	Staphylococcus aureus ssp. aureus 11819-97
1649.	Staphylococcus aureus ssp. aureus 71193
1650.	Staphylococcus aureus ssp. aureus COL
1651.	Staphylococcus aureus ssp. aureus ECT-R 2
1652.	Staphylococcus aureus ssp. aureus ED133
1653.	Staphylococcus aureus ssp. aureus ED98
1654.	Staphylococcus aureus ssp. aureus HO 5096 0412
1655.	Staphylococcus aureus ssp. aureus JH1
1656.	Staphylococcus aureus ssp. aureus JH9
1657.	Staphylococcus aureus ssp. aureus MSSA476
1658.	Staphylococcus aureus subsp. aureus MW2
1659.	Staphylococcus aureus ssp. aureus Mu3
1660.	Staphylococcus aureus ssp. aureus NCTC 8325
1661.	Staphylococcus aureus ssp. aureus ST398
1662.	Staphylococcus aureus ssp. aureus T0131
1663.	Staphylococcus aureus ssp. aureus TCH60
1664.	Staphylococcus aureus ssp. aureus TW20
1665.	Staphylococcus aureus ssp. aureus USA300_FPR3757
1666.	Staphylococcus aureus ssp. aureus USA300_TCH1516
1667.	Staphylococcus aureus ssp. aureus VC40
1668.	Staphylococcus aureus ssp. aureus str. JKD6008
1669.	Staphylococcus carnosus ssp. carnosus TM300
1670.	Staphylococcus epidermidis ATCC 12228
1671.	Staphylococcus epidermidis RP62A
1672.	Staphylococcus haemolyticus JCSC1435
1673.	Staphylococcus lugdunensis HKU09-01
1674.	Staphylococcus lugdunensis N920143
1675.	Staphylococcus pseudintermedius ED99
1676.	Staphylococcus pseudintermedius HKU10-03
1677.	Staphylococcus saprophyticus ssp. saprophyticus
1678.	Staphylococcus warneri SG1
1679.	Staphylothermus hellenicus DSM 12710

1680.	Staphylothermus marinus F1
1681.	Starkeya novella DSM 506
1682.	Stenotrophomonas maltophilia D457
1683.	Stenotrophomonas maltophilia JV3
1684.	Stenotrophomonas maltophilia K279a
1685.	Stenotrophomonas maltophilia R551-3
1686.	Stigmatella aurantiaca DW4/3-1
1687.	Streptobacillus moniliformis DSM 12112
1688.	Streptococcus agalactiae
1689.	Streptococcus agalactiae A909
1690.	Streptococcus agalactiae GD201008-001
1691.	Streptococcus agalactiae NEM316
1692.	Streptococcus agalactiae SA20-06
1693.	Streptococcus dysgalactiae ssp. equisimilis AC-2713
1694.	Streptococcus dysgalactiae ssp. equisimilis ATCC 12394
1695.	Streptococcus dysgalactiae ssp. equisimilis GGS_124
1696.	Streptococcus dysgalactiae ssp. equisimilis RE378
1697.	Streptococcus equi ssp. equi 4047
1698.	Streptococcus equi ssp. zooepidemicus
1699.	Streptococcus equi ssp. zooepidemicus str. MGCS10565
1700.	Streptococcus gallolyticus UCN34
1701.	Streptococcus gallolyticus ssp. gallolyticus ATCC 43143
1702.	Streptococcus gallolyticus ssp. gallolyticus ATCC BAA-2069
1703.	Streptococcus gordonii str. Challis sstr. CH1
1704.	Streptococcus infantarius ssp. infantarius CJ18
1705.	Streptococcus intermedius JTH08
1706.	Streptococcus macedonicus ACA-DC 198
1707.	Streptococcus mitis B6
1708.	Streptococcus mutans GS-5
1709.	Streptococcus mutans LJ23
1710.	Streptococcus mutans NN2025
1711.	Streptococcus mutans UA159

1712.	Streptococcus oralis Uo5
1713.	Streptococcus parasanguinis ATCC 15912
1714.	Streptococcus parasanguinis FW213
1715.	Streptococcus parauberis KCTC 11537
1716.	Streptococcus pasteurianus ATCC 43144
1717.	Streptococcus pneumoniae
1718.	Streptococcus pneumoniae 670-6B
1719.	Streptococcus pneumoniae 70585
1720.	Streptococcus pneumoniae G54
1721.	Streptococcus pneumoniae SPNA45
1722.	Streptococcus pneumoniae ST556
1723.	Streptococcus pneumoniae TCH8431/19A
1724.	Streptococcus pneumoniae Taiwan19F-14
1725.	Streptococcus pneumoniae gamPNI0373
1726.	Streptococcus pseudopneumoniae IS7493
1727.	Streptococcus pyogenes A20
1728.	Streptococcus pyogenes Alab49
1729.	Streptococcus pyogenes M1 476
1730.	Streptococcus pyogenes MGAS10270
1731.	Streptococcus pyogenes MGAS10394
1732.	Streptococcus pyogenes MGAS10750
1733.	Streptococcus pyogenes MGAS15252
1734.	Streptococcus pyogenes MGAS1882
1735.	Streptococcus pyogenes MGAS2096
1736.	Streptococcus pyogenes MGAS315 s. M3
1737.	Streptococcus pyogenes MGAS5005
1738.	Streptococcus pyogenes MGAS6180
1739.	Streptococcus pyogenes MGAS9429
1740.	Streptococcus pyogenes NZ131
1741.	Streptococcus pyogenes SSI-1
1742.	Streptococcus pyogenes str. Manfredo
1743.	Streptococcus pyogenes MGAS8232 s. M18

1744.	Streptococcus pyogenes SF370 s. M1
1745.	Streptococcus salivarius CCHSS3
1746.	Streptococcus salivarius JIM8777
1747.	Streptococcus sanguinis SK36
1748.	Streptococcus suis 05ZYH33
1749.	Streptococcus suis 98HAH33
1750.	Streptococcus suis A7
1751.	Streptococcus suis BM407
1752.	Streptococcus suis D12
1753.	Streptococcus suis D9
1754.	Streptococcus suis GZ1
1755.	Streptococcus suis JS14
1756.	Streptococcus suis P1/7
1757.	Streptococcus suis S735
1758.	Streptococcus suis SC84
1759.	Streptococcus suis SS12
1760.	Streptococcus suis ST1
1761.	Streptococcus suis ST3
1762.	Streptococcus thermophilus CNRZ1066
1763.	Streptococcus thermophilus JIM 8232
1764.	Streptococcus thermophilus LMD-9
1765.	Streptococcus thermophilus MN-ZLW-002
1766.	Streptococcus thermophilus ND03
1767.	Streptococcus uberis 0140J
1768.	Streptomyces avermitilis
1769.	Streptomyces bingchenggensis BCW-1
1770.	Streptomyces cattleya NRRL 8057
1771.	Streptomyces cattleya NRRL 8057 = DSM 46488
1772.	Streptomyces coelicolor A32
1773.	Streptomyces davawensis JCM 4913
1774.	Streptomyces flavogriseus ATCC 33331
1775.	Streptomyces griseus ssp. griseus NBRC 13350

1776.	Streptomyces hygroscopicus subsp. jinggangensis 5008
1777.	Streptomyces scabiei 87.22
1778.	Streptomyces sp. SirexAA-E
1779.	Streptomyces venezuelae ATCC 10712
1780.	Streptomyces violaceusniger Tu 4113
1781.	Streptosporangium roseum DSM 43021
1782.	Sulfobacillus acidophilus TPY
1783.	Sulfolobus acidocaldarius DSM 639
1784.	Sulfolobus acidocaldarius N8
1785.	Sulfolobus acidocaldarius Ron12/I
1786.	Sulfolobus islandicus HVE10/4
1787.	Sulfolobus islandicus L.D.8.5
1788.	Sulfolobus islandicus L.S.2.15
1789.	Sulfolobus islandicus M.14.25
1790.	Sulfolobus islandicus M.16.27
1791.	Sulfolobus islandicus M.16.4
1792.	Sulfolobus islandicus Y.G.57.14
1793.	Sulfolobus islandicus Y.N.15.51
1794.	Sulfolobus solfataricus 98/2
1795.	Sulfolobus solfataricus P2
1796.	Sulfolobus tokodaii
1797.	Sulfuricurvum kujiense DSM 16994
1798.	Sulfurihydrogenibium azorense Az-Fu1
1799.	Sulfurihydrogenibium sp. YO3AOP1
1800.	Sulfurimonas autotrophica DSM 16294
1801.	Sulfurospirillum barnesii SES-3
1802.	Sulfurospirillum deleyianum DSM 6946
1803.	Sulfurovum sp. NBC37-1
1804.	Symbiobacterium thermophilum
1805.	Synechococcus elongatus PCC 6301
1806.	Synechococcus elongatus PCC 7942
1807.	Synechococcus sp. CC9311

1808.	Synechococcus sp. CC9605
1809.	Synechococcus sp. CC9902
1810.	Synechococcus sp. PCC 6312
1811.	Synechococcus sp. PCC 7002
1812.	Synechococcus sp. PCC 7502
1813.	Synechococcus sp. WH 7803
1814.	Synechococcus sp. WH 8102
1815.	Synechocystis sp. PCC6803
1816.	Synechocystis sp. PCC 6803 sstr. GT-S
1817.	Synechocystis sp. PCC 6803 Moscow wildtype
1818.	Synechocystis sp. PCC 6803 sstr. GT-I
1819.	Synechocystis sp. PCC 6803 sstr. PCC-N
1820.	Synechocystis sp. PCC 6803 sstr. PCC-P
1821.	Syntrophobacter fumaroxidans MPOB
1822.	Syntrophobotulus glycolicus DSM 8271
1823.	Syntrophomonas wolfei ssp. wolfei str. Goettingen
1824.	Syntrophothermus lipocalidus DSM 12680
1825.	Syntrophus aciditrophicus SB
1826.	Tannerella forsythia ATCC 43037
1827.	Taylorella asinigenitalis MCE3
1828.	Taylorella equigenitalis ATCC 35865
1829.	Taylorella equigenitalis MCE9
1830.	Tepidanaerobacter acetatoxydans Re1
1831.	Tepidanaerobacter sp. Re1
1832.	Teredinibacter turnerae T7901
1833.	Terriglobus roseus DSM 18391
1834.	Terriglobus saanensis SP1PR4
1835.	Tetragenococcus halophilus NBRC 12172
1836.	Thalassobaculum sp. L2
1837.	Thauera sp. MZ1T
1838.	Thermacetogenium phaeum DSM 12270
1839.	Thermaerobacter marianensis DSM 12885

1840.	Thermanaerovibrio acidaminovorans DSM 6589
1841.	Thermincola sp. JR
1842.	Thermoanaerobacter brockii ssp. finnii Ako-1
1843.	Thermoanaerobacter italicus Ab9
1844.	Thermoanaerobacter mathranii ssp. mathranii str. A3
1845.	Thermoanaerobacter pseudethanolicus ATCC 33223
1846.	Thermoanaerobacter sp. X513
1847.	Thermoanaerobacter sp. X514
1848.	Thermoanaerobacter tengcongensis
1849.	Thermoanaerobacter wiegelii Rt8.B1
1850.	Thermoanaerobacterium saccharolyticum JW/SL-YS485,
1851.	Thermoanaerobacterium thermosaccharolyticum DSM 571
1852.	Thermoanaerobacterium thermosaccharolyticum M0795
1853.	Thermoanaerobacterium xylanolyticum LX-11
1854.	Thermobacillus composti KWC4
1855.	Thermobifida fusca YX
1856.	Thermobispora bispora DSM 43833
1857.	Thermococcus barophilus MP
1858.	Thermococcus gammatolerans EJ3
1859.	Thermococcus kodakaraensis KOD1
1860.	Thermococcus onnurineus NA1
1861.	Thermococcus sibiricus MM 739
1862.	Thermococcus sp. 4557
1863.	Thermococcus sp. AM4
1864.	Thermococcus sp. CL1
1865.	Thermocrinis albus DSM 14484
1866.	Thermodesulfatator indicus DSM 15286
1867.	Thermodesulfobacterium sp. OPB45
1868.	Thermodesulfobium narugense DSM 14796
1869.	Thermodesulfovibrio yellowstonii DSM 11347
1870.	Thermofilum pendens Hrk 5
1871.	Thermogladius sp. 1633

1872.	Thermomicrobium roseum DSM 5159
1873.	Thermomonospora curvata DSM 43183
1874.	Thermoplasma acidophilum
1875.	Thermoplasma volcanium
1876.	Thermoproteus neutrophilus V24Sta
1877.	Thermoproteus tenax Kra 1
1878.	Thermoproteus uzoniensis 768-20
1879.	Thermosediminibacter oceani DSM 16646
1880.	Thermosipho africanus TCF52B
1881.	Thermosipho melanesiensis BI429
1882.	Thermosphaera aggregans DSM 11486
1883.	Thermosynechococcus elongatus BP-1
1884.	Thermotoga lettingae TMO
1885.	Thermotoga maritima
1886.	Thermotoga naphthophila RKU-10
1887.	Thermotoga neapolitana DSM 4359
1888.	Thermotoga petrophila RKU-1
1889.	Thermotoga sp. RQ2
1890.	Thermotoga thermarum DSM 5069
1891.	Thermotogales bacterium TBF 19.5.1
1892.	Thermovibrio ammonificans HB-1
1893.	Thermovirga lienii DSM 17291
1894.	Thermus oshimai JL-2
1895.	Thermus scotoductus SA-01
1896.	Thermus sp. CCB_US3_UF1
1897.	Thermus thermophilus HB27
1898.	Thermus thermophilus HB8.
1899.	Thermus thermophilus JL-18
1900.	Thioalkalimicrobium cyclicum ALM1
1901.	Thioalkalivibrio nitratireducens DSM 14787
1902.	Thioalkalivibrio sp. K90mix
1903.	Thioalkalivibrio sulfidophilus HL-EbGr7

1904.	Thiobacillus denitrificans ATCC 25259
1905.	Thiocystis violascens DSM 198
1906.	Thioflavicoccus mobilis 8321
1907.	Thiomicrospira crunogena XCL-2
1908.	Thiomicrospira denitrificans ATCC 33889
1909.	Thiomonas intermedia K12
1910.	Thiomonas sp. 3As
1911.	Tistrella mobilis KA081020-065
1912.	Tolumonas auensis DSM 9187
1913.	Treponema azotonutricium ZAS-9
1914.	Treponema brennaborense DSM 12168
1915.	Treponema denticola ATCC 35405
1916.	Treponema pallidum
1917.	Treponema pallidum ssp. pallidum DAL-1
1918.	Treponema pallidum ssp. pallidum SS14
1919.	Treponema pallidum ssp. pallidum str. Chicago
1920.	Treponema pallidum ssp. pallidum str. Mexico A
1921.	Treponema pallidum ssp. pertenue str. CDC2
1922.	Treponema pallidum ssp. pertenue str. Gauthier
1923.	Treponema pallidum ssp. pertenue str. SamoaD
1924.	Treponema paraluiscuniculi Cuniculi A
1925.	Treponema primitia ZAS-2
1926.	Treponema succinifaciens DSM 2489
1927.	Trichodesmium erythraeum IMS101
1928.	Tropheryma whipplei TW08/27
1929.	Tropheryma whipplei str. Twist
1930.	Truepera radiovictrix DSM 17093
1931.	Tsukamurella paurometabola DSM 20162
1932.	Turneriella parva DSM 21527
1933.	Ureaplasma parvum sv. 3 str. ATCC 27815
1934.	Ureaplasma urealyticum
1935.	Ureaplasma urealyticum sv. 10 str. ATCC 33699

1936.	Variovorax paradoxus EPS
1937.	Veillonella parvula DSM 2008
1938.	Verminephrobacter eiseniae EF01-2
1939.	Verrucosispora maris AB-18-032
1940.	Vibrio parahaemolyticus BB22OP
1941.	Vibrio parahaemolyticus BB22OP
1942.	Vulcanisaeta distributa DSM 14429
1943.	Vulcanisaeta moutnovskia 768-28
1944.	Waddlia chondrophila WSU 86-1044
1945.	Weeksella virosa DSM 16922
1946.	Weissella koreensis KACC 15510
1947.	Wigglesworthia brevipalpis
1948.	Wigglesworthia glossinidia endosymbiont of Glossina morsitans
1949.	Wolbachia endosymbiont of Culex quinquefasciatus Pel
1950.	Wolbachia endosymbiont of Drosophila melanogaster
1951.	Wolbachia endosymbiont of Onchocerca ochengi
1952.	Wolbachia endosymbiont str TRS of Brugia malayi
1953.	Wolbachia sp. wRi
1954.	Wolinella succinogenes
1955.	Xanthobacter autotrophicus Py2
1956.	Xanthomonas albilineans
1957.	Xanthomonas axonopodis Xac29-1
1958.	Xanthomonas axonopodis pv. citri str. 306
1959.	Xanthomonas axonopodis pv. citrumelo F1
1960.	Xanthomonas campestris pv. campestris str. 8004
1961.	Xanthomonas campestris
1962.	Xanthomonas campestris pv. campestris str.B100
1963.	Xanthomonas campestris pv. raphani 756C
1964.	Xanthomonas campestris pv. vesicatoria str. 85-10
1965.	Xanthomonas citri ssp. citri Aw12879
1966.	Xanthomonas oryzae pv. oryzae KACC 10331
1967.	Xanthomonas oryzae pv. oryzae MAFF 311018

1968.	Xanthomonas oryzae pv. oryzae PXO99A
1969.	Xenorhabdus bovienii SS-2004
1970.	Xenorhabdus nematophila ATCC 19061
1971.	Xenorhabdus nematophila ATCC 19061
1972.	Xylanimonas cellulosilytica DSM 15894
1973.	Xylella fastidiosa
1974.	Xylella fastidiosa M12
1975.	Xylella fastidiosa M23
1976.	Xylella fastidiosa Temecula1
1977.	Xylella fastidiosa ssp. fastidiosa GB514
1978.	Yersinia enterocolitica ssp. enterocolitica 8081
1979.	Yersinia enterocolitica ssp. palearctica Y11
1980.	Yersinia pestis A1122
1981.	Yersinia pestis D106004
1982.	Yersinia pestis D182038
1983.	Yersinia pestis KIM 10
1984.	Yersinia pestis Nepal516
1985.	Yersinia pestis Pestoides F
1986.	Yersinia pestis Z176003
1987.	Yersinia pestis biovar Medievalis str. Harbin 35
1988.	Yersinia pestis biovar Microtus str. 91001
1989.	Yersinia pestis CO92
1990.	Yersinia pseudotuberculosis IP 31758
1991.	Yersinia pseudotuberculosis IP32953
1992.	Yersinia pseudotuberculosis PB1/+
1993.	Yersinia pseudotuberculosis YPIII
1994.	Zobellia galactanivorans
1995.	Zunongwangia profunda SM-A87
1996.	Zymomonas mobilis ssp. mobilis ATCC 10988
1997.	Zymomonas mobilis ssp. mobilis ATCC 29191
1998.	Zymomonas mobilis ssp. mobilis NCIB 11163
1999.	Zymomonas mobilis ss. mobilis ZM4

2000.	Zymomonas mobilis ssp. pomaceae ATCC 29192
